# Homoharringtonine: mechanisms, clinical applications and research progress

**DOI:** 10.3389/fonc.2025.1522273

**Published:** 2025-01-30

**Authors:** Wen Wang, Lan He, Ting Lin, Fulan Xiang, Yibin Wu, Fangliang Zhou, Yingchun He

**Affiliations:** ^1^ Graduate School, Hunan University of Chinese Medicine, Changsha, China; ^2^ Hunan Provincial Engineering and Technological Research Center for Prevention and Treatment of Ophthalmology and Otolaryngology Diseases with Chinese Medicine and Protecting Visual Function, Hunan University of Chinese Medicine, Changsha, China; ^3^ Hunan Provincial Key Laboratory for the Prevention and Treatment of Ophthalmology and Otolaryngology Diseases with Traditional Chinese Medicine, Hunan University of Chinese Medicine, Changsha, China; ^4^ Medical School, Hunan University of Chinese Medicine, Changsha, China

**Keywords:** homoharringtonine, hematological disorders, antitumor activity, pharmacological mechanisms, clinical applications, cancer treatment

## Abstract

Homoharringtonine is a natural alkaloid with significant pharmacological potential that has demonstrated promising efficacy in the treatment of hematological malignancies in recent years. This article systematically reviews the pharmacological mechanisms of Homoharringtonine, focusing on its key roles in inducing apoptosis, inhibiting cell cycle progression, and reducing cell migration and invasion. Additionally, HHT exhibits multiple biological activities, including immunomodulation, antiviral effects, and anti-fibrotic properties, with recent studies also revealing its potential neuroprotective functions. In clinical trials, Homoharringtonine has demonstrated promising efficacy in the treatment of hematological malignancies, particularly in various types such as acute myeloid leukemia and chronic myeloid leukemia. Despite the significant antitumor effects observed in clinical applications, its low bioavailability and potential side effects remain major challenges that limit its widespread use. This article details the latest research advancements aimed at enhancing the bioavailability of Homoharringtonine, including various drug delivery systems such as nanoparticles and liposomes, as well as chemical modification strategies. These approaches not only improve HHT’s bioavailability *in vivo* but also enhance its targeting ability while reducing toxicity to normal cells. Furthermore, the combination of HHT with other drugs presents broader prospects for clinical treatment. By exploring the diverse pharmacological activities of Homoharringtonine in depth, this article aims to provide a foundation for developing novel therapeutic approaches based on natural products, thereby advancing HHT’s application research in cancer treatment and other fields.

## 1 Introduction

**Figure 1:**
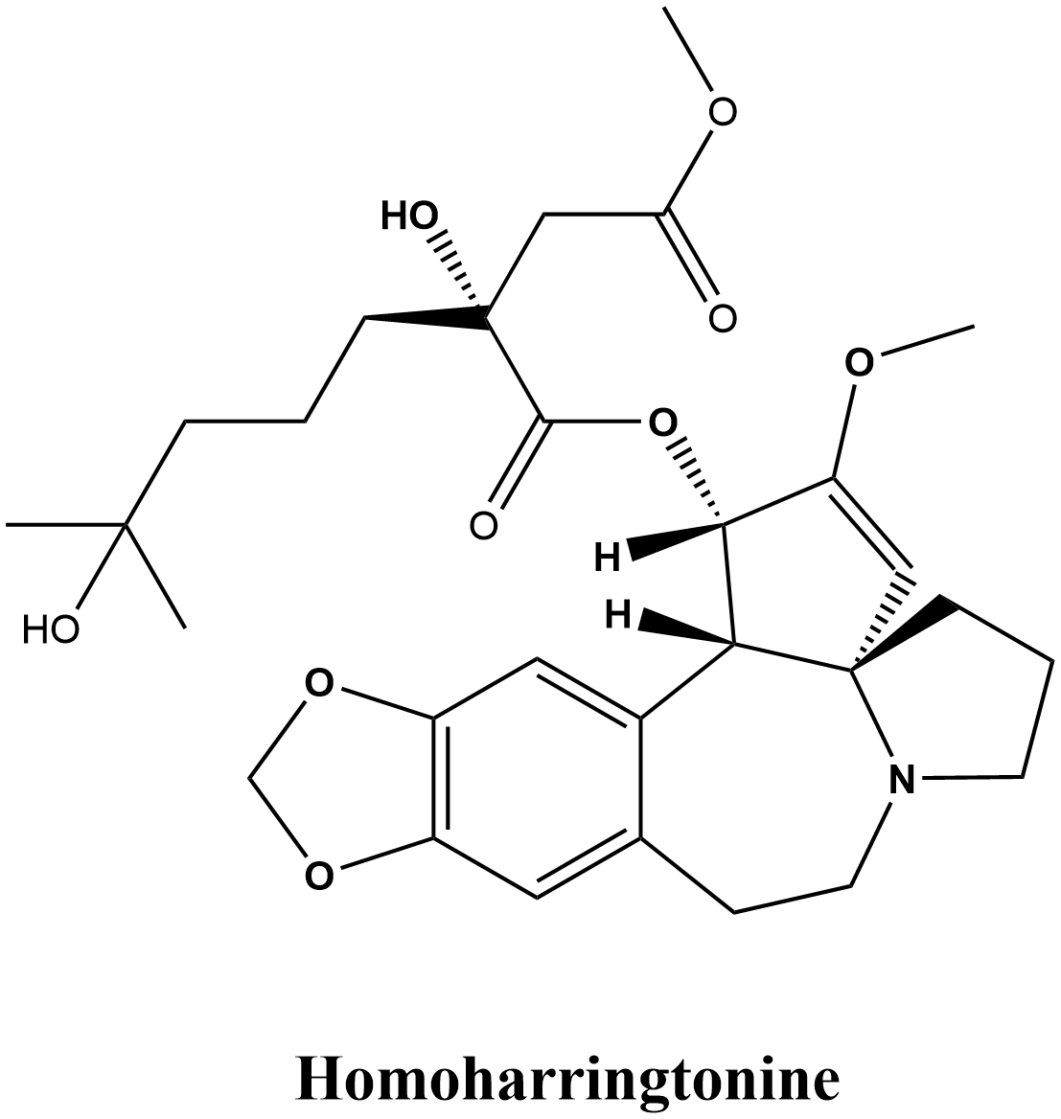
Chemical structure of HHT.

Homoharringtonine (HHT) is an alkaloid extracted from *Cephalotaxus fortunei* Hook. and its related species, celebrated for its notable anticancer properties, particularly in leukemia treatment (1, 2). Its therapeutic history can be traced back to traditional medicine’s knowledge of the *Cephalotaxus* species, which predominantly thrive in East Asia within the Cephalotaxaceae family. Various parts of *Cephalotaxus* plants, including the bark, leaves, and seeds, possess medicinal properties and have been traditionally utilized to treat diverse conditions such as malignant tumors, cough, fever, injuries, scabies, specific skin ailments, and vaginal cysts (3). Clinical studies have substantiated the efficacy of *Cephalotaxus* extracts and compounds in the treatment of diseases such as malignancies, lung cancer, and acute leukemia (4, 5). As early as 1969, Powell et al. (6–8) identified four alkaloids derived from Cephalotaxus plants—Harringtonine, Homoharringtonine, Isoharringtonine, and Deoxyharringtonine—that prevent the proliferation of mouse leukemia cells, and determined the structure of HHT (C_29_H_39_NO_9_, **Figure 1**). Chinese researchers used HHT to treat leukemia clinically in 1977 after demonstrating that it significantly affects human non-lymphocytic leukemia (9). Entering the 1990s, the pharmacological effects of HHT were increasingly validated, gradually establishing it as an important anti-tumor agent (10). In the 2000s, researchers began to explore various new applications of HHT, discovering its potential use in treating other malignancies such as liver cancer (11) and breast cancer (12). In 2012, the U.S. Food and Drug Administration (FDA) approved HHT (omacetaxine mepesuccinate) for treating chronic myeloid leukemia (CML) (13). In the 2010s, combination therapies involving HHT became widely adopted (14, 15). (The plant name *Cephalotaxus fortunei* Hook. was verified with the World Flora Online database on August 18, 2024.)

Studies have shown that HHT exerts significant therapeutic effects in hematological diseases by blocking nascent peptide chain elongation and inhibiting protein synthesis (16). It also functions by regulating inflammatory factors, enzymes, and apoptosis-related proteins. As research into HHT’s mechanisms has deepened, its applications have expanded beyond hematological disorders into other pathological areas. In addition to its antitumor efficacy, HHT demonstrates therapeutic potential in diseases such as inflammation, viral infections, and fibrosis by modulating multiple signaling pathways (17–22). This review summarizes HHT’s pharmacological mechanisms, therapeutic potential, adverse effects (23), and strategies to enhance bioavailability (24).

**Figure 2:**
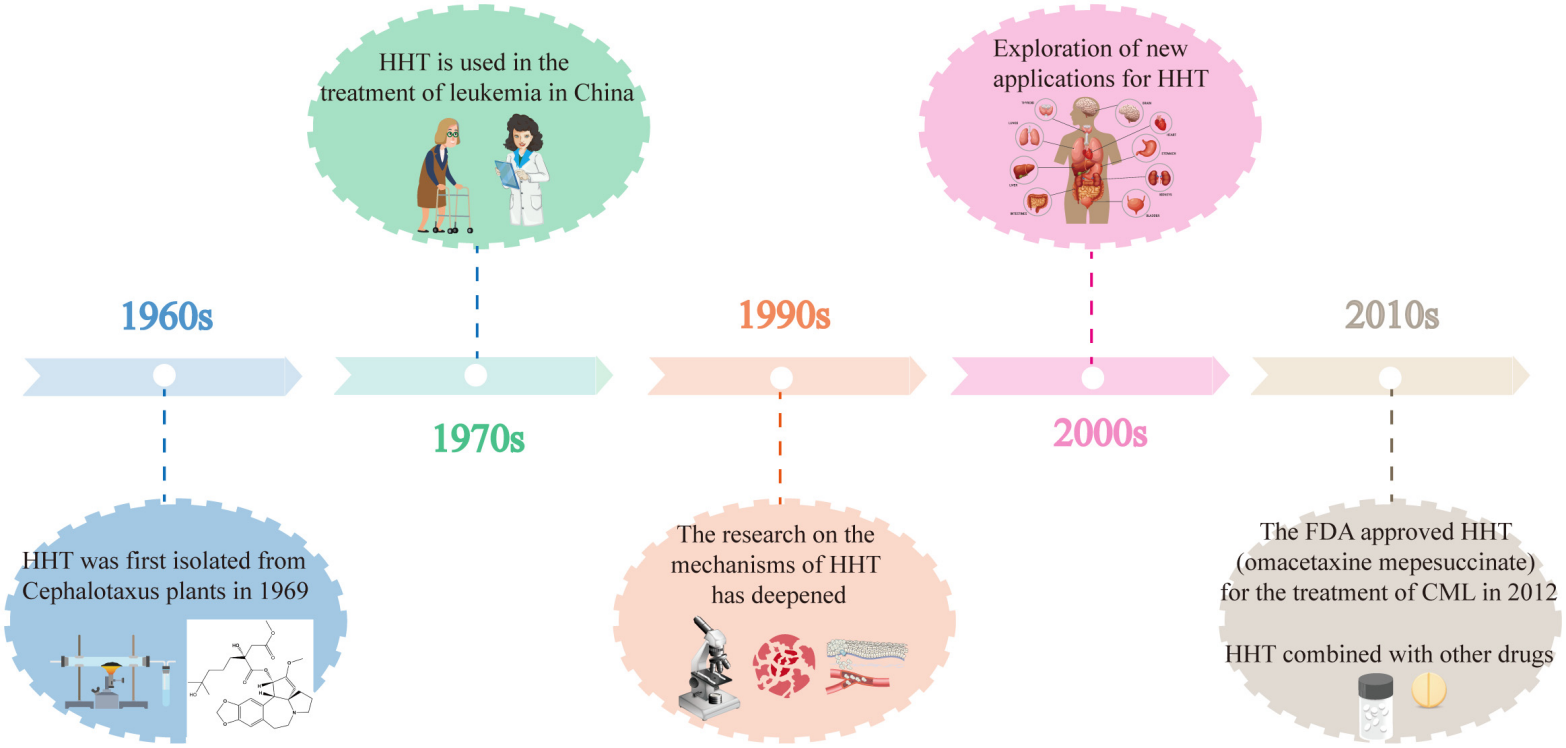
The key developments in the history of HHT.

**Figure 2** illustrates the key developments in the history of HHT, highlighting important milestones such as its discovery, clinical applications, and research advancements.

## 2 Impact of HHT on hematological malignancies and its mechanisms of action

### 2.1 Leukemia

Leukemia, a malignancy affecting hematopoietic stem cells, is linked to exposure to benzene, ionizing radiation, and genetic mutations. This disease presents with severe infections, anemia, and bleeding, resulting from the abnormal expansion of hematopoietic stem cells within the bone marrow, disrupting normal hematopoiesis (25). HHT acts as an anti-leukemia drug for leukemia treatment by mechanisms that are multifarious. Previous literature has reported that HHT can influence the expression levels of transcription factors, such as nuclear factor kappa B (NF-κB) (26), Forkhead box protein M1 (FOXM1) (27), specificity protein 1 (SP1) (28), the B-cell lymphoma-2 (Bcl-2)/Bcl-2-associated X protein (Bax) complex (29), the caspase family (30), telomerase (31), CDK2 (32), myosin-9 (33), and p53 (14). Furthermore, it can inhibit protein synthesis, block the cell cycle process, and disturb the activity of cell signaling pathways, resulting in many impacts on the proliferation and apoptosis of leukemia cells.

HHT functions as a translation inhibitor, acting by interacting with the ribosomal A site to inhibit the initiation and initial elongation steps of translation. This process induces cancer cell death (34–36). NF-κB-repressing factor (NKRF) is a transcriptional repressor of NF-κB, interacting with NF-κB to suppress its activity (37). The binding of HHT to NKRF disrupts the translocation of p65 and its binding to the *MYC* promoter, resulting in the downregulation of MYC transcriptional expression, subsequently suppressing the growth of t (7, 20) acute myeloid leukemia (AML) cells (26). In AML and chronic myeloma leukemia (CML) cell lines, HHT upregulates the expression of the cytoskeletal protein myosin-9 in a time-dependent manner (33). This upregulation arrests cells in the S and G2/M phases and increases the sensitivity of leukemia cells to HHT’s cytotoxic effects, ultimately inhibiting leukemic cell growth. Moreover, HHT enhances the binding affinity between CDK2 and tripartite motif 21 (Trim21) by specifically targeting the interaction between CDK2 and Cyclin A. This leads to the autophagic degradation of CDK2, thereby modulating the cell cycle of leukemia cells. Consequently, HHT significantly inhibits leukemia progression in both leukemia mouse models and human primary leukemia cells (32). FOXM1, an oncogenic transcription factor and member of the Forkhead family, is also a known target of HHT. FOXM1 is involved in cell cycle regulation, proliferation, invasion, vascular invasion, angiogenesis, oxidative stress, and inflammation (38). Targeting FOXM1 with HHT sensitizes K562 leukemia cells to the drug (27). Another study reported that HHT’s antitumor function is associated with the competitive action in which HHT can bind to SP1, which is one of the key transcriptional activators, in the TET1 promoter. SP1 is an essential transcriptional activator (39, 40). TET1 is a 5-methylcytosine hydroxylase which is critical for demethylation (41). HHT functions in the SP1/TET1/5hmC/FLT3 axis by inhibiting SP1-mediated TET1 transcription, which regulates the DNA epigenome of AML and modulates the growth of FLT3-mutant AML cells (28). Moreover, telomerase activity is essential for telomerase immortalize tumor cells and transform them to malignant status (42, 43). Around 80% of acute leukemia cells have increased telomerase activity (44, 45). Another research study showed that HHT induced apoptosis in human leukemic HL-60 cells by inhibiting their telomerase, indicating that telomerase may be a possible therapeutic target of leukemia treatment (31). By triggering the mTOR signaling pathway, HHT also suppresses the expression of the anti-apoptotic B-cell lymphoma 6 protein (BCL-6), which causes leukemic K562 cells to undergo apoptosis (46). HHT exerts a dual effect on K562 cells: it induces apoptosis through the activation of Caspase-3 and simultaneously triggers autophagy under continuous exposure to HHT. When combined with autophagy inhibitors such as 3-methyladenine (3-MA), the cytotoxic effects of HHT are significantly enhanced, and the inhibition of autophagy further potentiates HHT-induced apoptosis (47). The above results show that HHT is able to exert anti-leukemic effects by regulating the DNA epigenome, blocking the cell cycle, and inducing apoptosis.

### 2.2 Other blood disorders

In a few studies, HHT has been shown to have some potential effects in multiple myeloma (MM) and lymphoma treatment. Studies have demonstrated that HHT markedly enhances the anti-myeloma efficacy of BTZ in both *in vitro* multiple myeloma (MM) cell models and *in vivo* mouse xenotransplantation models through inhibition of the NF-κB signaling pathway (48). Furthermore, HHT exerts anti-tumor effects by inducing mitochondrial autophagy and mitochondrial dysfunction, with Parkin-dependent autophagy playing a crucial role in this process (49).

Approximately 40% of all malignant lymphoid neoplasms consist of diffuse large B-cell lymphoma (DLBCL), the most prevalent form of malignant lymphoma (50). A recent study has reported that HHT can induce apoptosis in DLBCL cells, which is done by decreasing the expression levels of the anti-apoptotic protein myeloid cell leukemia-1 (Mcl-1) and B-cell lymphoma-2 (Bcl-2) (51).

When HHT was co-administered with bortezomib (BTZ), it effectively reduced the expression of the anti-apoptotic protein Mcl-1, increased the levels of the pro-apoptotic protein NADPH oxidase activator (Noxa), and activated the pro-apoptotic protein Bcl-2 homologous antagonist/killer (Bak) (19). Either HHT, or HHT in combination with curcumin, was able to inhibit the growth and angiogenesis of lymphoma cells by targeting the Vascular Endothelial Growth Factor/Protein Kinase B (VEGF/AKT) signaling pathway (29).

**Figure 3:**
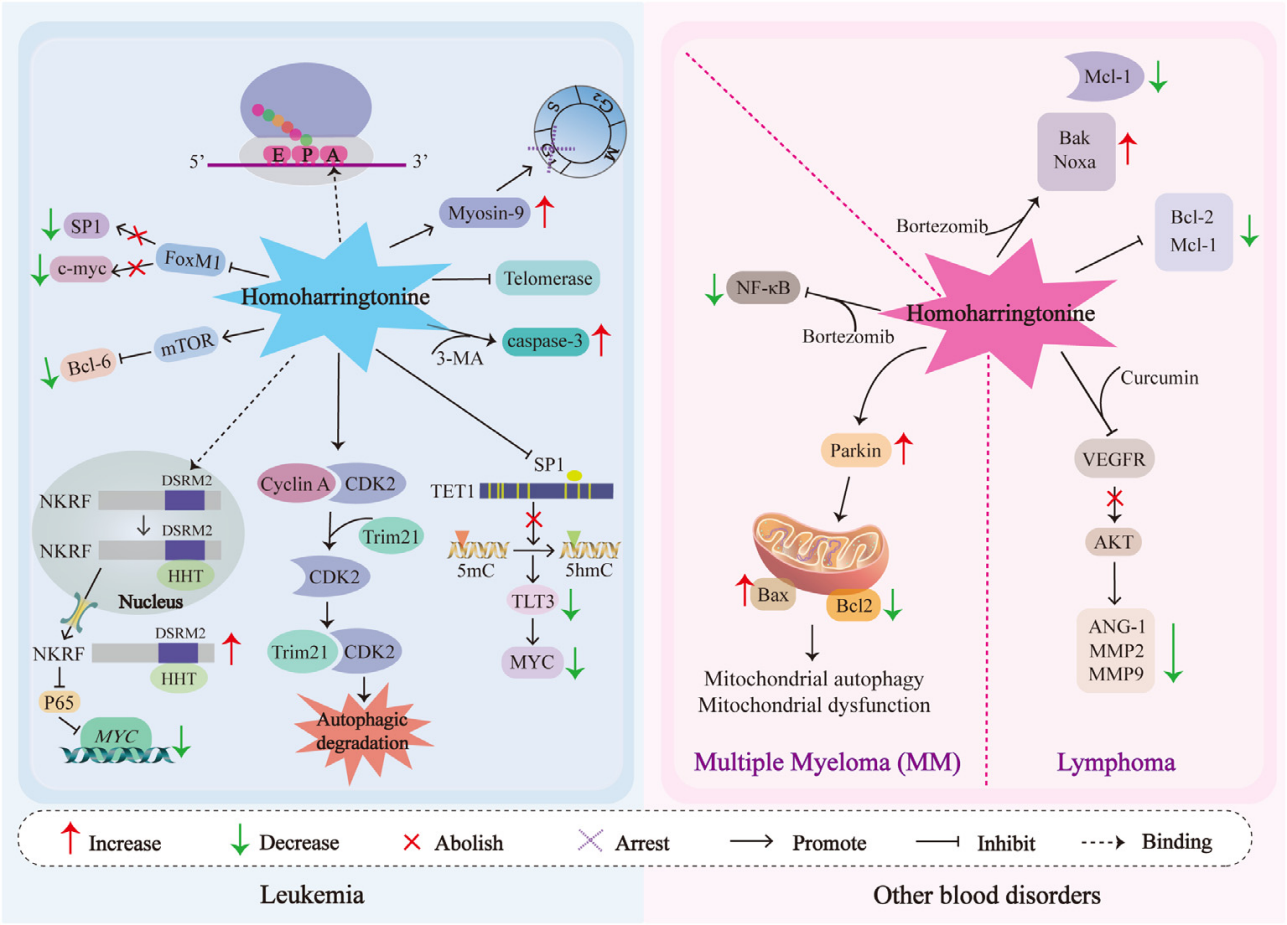
The schematic diagram of signal transduction of the pathways affected by HHT in cytotoxicity, anti-proliferation, cell cycle arrest and antimetastasis in blood tumor cells is illustrated. NKRF, NF-κB-repressing factor; MYC, Myelocytomatosis oncogene; c-Myc, Cellular Myc; CDK2, Cyclin-dependent kinase 2; Trim21, tripartite motif 21; FOXM1, Forkhead box protein M1; SP1, Specificity protein 1; TET1, Ten-Eleven Translocation 1; 5hmC, 5-hydroxymethylcytosine; FLT3, FMS-like tyrosine kinase 3; mTOR, Mechanistic Target of Rapamycin; BCL-6, B-cell lymphoma 6; Bcl-2, B-cell lymphoma-2; Mcl-1, Myeloid cell leukemia-1; Noxa, NADPH oxidase activator; Bak, Bcl-2 homologous antagonist/killer; Caspases-3, Cysteinyl aspartate-specific protease-3; VEGF, Vascular Endothelial Growth Factor; AKT, Protein Kinase B; MMP2/9, Matrix Metalloproteinase2/9; ANG-1, Angiopoietin-1; Bax, Bcl-2-associated X protein; 3-MA, 3-methyladenine.

**Figure 3** shows the mechanisms of action of HHT in hematological tumors.

## 3 Impact of HHT on cancer and its mechanisms of action

### 3.1 Colorectal cancer

In the treatment of colorectal cancer, HHT exerts its therapeutic effects by regulating proliferation, apoptosis, and cell cycle-related signaling pathways. Ephrin type-B receptor 4 (EphB4) belongs to the Ephrin-B receptor family of receptor tyrosine kinases, which are involved in cell adhesion, migration, and angiogenesis, and EphB4 overexpression is closely associated with tumor invasion, metastasis, and prognosis (52, 53). HHT targets EphB4, inhibiting the activation of the Mitogen-Activated Protein Kinase/Extracellular Signal-Regulated Kinase 1/2 (MAPK/ERK1/2) and Phosphoinositide 3-Kinase (PI3K)/AKT pathways, while regulating the expression of cell cycle-related proteins (such as cyclin A2 and CDC2) and apoptosis-related proteins (including Bcl-2/Bax, Mcl-1, Bad, and caspases-3, 7, and 9), thereby effectively suppressing the progression of colorectal cancer cells (LoVo) (20). The PI3K/AKT signaling pathway is intricately involved in tumorigenesis, cellular proliferation, metastasis, apoptosis, epithelial-mesenchymal transition (EMT), metabolism, and chemoresistance (54–57). HHT modulates colorectal cancer cell proliferation, apoptosis, and xenograft growth by targeting the PI3K/AKT/mTOR signaling pathway (21, 58). In addition, tumor necrosis factor-related apoptosis-inducing ligand (TRAIL) promotes apoptosis by binding to TRAIL receptors on the surface of tumor cells (59, 60). HHT, as a sensitizer of TRAIL, has demonstrated significant potential in anticancer therapy. HHT markedly enhances the anticancer efficacy of TRAIL by downregulating the expression of anti-apoptotic proteins such as Mcl-1 and cFLIP, activating pro-apoptotic signaling pathways including JNK and p38, and synergizing with TRAIL to induce necroptotic cell death via the Receptor-Interacting Protein Kinase 1/Receptor-Interacting Protein Kinase 3/Mixed-Lineage Kinase Domain-Like Protein (RIPK1/RIPK3/MLKL) signaling pathway (61–63). In summary, HHT not only inhibits tumor cell proliferation, migration, and invasion, but also overcomes drug resistance in cancer cells, thereby promoting tumor cell death when combined with TRAIL.

### 3.2 Liver cancer

Yang et al. (64) demonstrated the anti-fibrotic and anti-tumor effects of HHT through investigations employing a subcutaneous xenograft tumor model and a carbon tetrachloride (CCl4)-induced liver fibrosis model. The Hippo pathway regulates cell proliferation and organ size balance, crucial for maintaining normal tissue morphology and development (65). Activation of the Hippo pathway by HHT inhibits the transition from the G1 phase to the S phase of the cell cycle, promoting cell apoptosis and impacting cellular proliferation, migration, and invasion (66). EMT induced by β-catenin is a prerequisite for cell migration (67, 68). The expressions of matrix metalloproteinases (MMPs), EphB4 and β-catenin were downregulated in tumor tissues from HepG2 cells and Xenograft model tissues treated with HHT (69, 70). Furthermore, HHT suppressed the PI3K/AKT/Glycogen Synthase Kinase 3β (GSK3β) signaling pathway and decreased snail family transcriptional repressor 2 (Slug) expression, ultimately suppressing EMT in hepatocellular carcinoma cells (71). Ataxia-telangiectasia mutated (ATM) is essential for the repair of double-stranded DNA breaks (72). In PLC5 hepatocellular carcinoma cells treated with HHT, DNA damage is induced. Upon sensing this damage, ATM is activated, which subsequently leads to p53 activation. p53 activates p21, which blocks the cyclin A/cyclin-dependent kinase 2 (CDK2) complex, collectively driving cellular apoptosis, arresting cell cycle progression, and inhibiting cellular proliferation (73). Additionally, studies have shown that HHT induces a mitochondria-mediated intrinsic apoptotic pathway through the upregulation of transforming growth factor-β (TGF-β) expression, TNF, Fas cell surface death receptor (FAS), p38 mitogen-activated protein kinase (p38MAPK) and p53 in a human hepatoma cell line QGY-7703 (11).

### 3.3 Lung cancer

Research results reveal that the anti-cancer efficacy of HHT is contingent upon its capability to stop the proliferation and viability of tumor cells through inhibiting protein synthesis, decreasing oncogenic expression of proteins in cancer cells, inducing apoptosis, and interfering with signal pathways. Nuclear factor erythroid 2-related factor 2 (NRF2) is essential for modulating the antioxidant and cellular defense responses through its role as a transcription factor (74). Inhibition of NRF2 transcription in HHT-treated A549 lung cancer cells was associated with increased sensitivity to the anticancer drug etoposide. This suggests that HHT may potentiate the efficacy of etoposide against A549 cells by suppressing NRF2 expression and disrupting NRF2-mediated antioxidant and cellular defense mechanisms (75). Transmembrane protein 16A (TMEM16A) is a membrane protein that functions as an ion channel and plays a role in regulating various physiological processes, and its high expression is linked to the proliferation, invasion, and metastasis of tumor cells (76). TMEM16A has been reported to promote tumorigenesis and cancer progression by activating the MAPK signaling pathway (77). HHT downregulated TMEM16A expression in a dose-dependent manner in lung cancer LA795 cell lines, which in turn reduced the phosphorylation of MEK1/2 and ERK1/2 in the MAPK pathway, affecting tumor cell proliferation, invasion, and cell cycle (78). The Kirsten rat sarcoma viral oncogene homolog (KRAS) is subject to frequent mutations in various types of cancer, especially in non-small cell lung cancer (NSCLC), where KRAS mutations dysregulate downstream cell signaling pathways such as PI3K/AKT and MAPK/ERK, resulting in abnormal activation of cellular proliferation and survival mechanisms (79, 80). It was shown that, in murine lung tumor models with Kras mutations G12D and G12C, HHT showed remarkable therapeutic efficiency and immunomodulatory effects. After treatment with HHT, interleukin-12 production in splenocytes was significantly decreased compared to the control group. HHT treatment also induced a notable increase in CD80, CD86 and CD69 expression on B220+ B cells. These data suggest that HHT acts by modulating immune cell biology and anti-tumor activity in KRAS-mutated lung tumors (81). Interleukin-6 (IL-6) is an oncogenic mediator of utmost importance that mediates immune and inflammatory responses, activates Signal Transducer and Activator of Transcription 3 (STAT3) in tumor cells, and its effects on oncogenesis, tumor immunosuppression, tumor angiogenesis, and metastasis are well established (82, 83). HHT treatment can affect IL-6 signaling by targeting Janus kinase 1 (JAK1) and STAT3, inhibit STAT3 nuclear translocation and transcriptional activity by antagonizing IL-6-induced STAT3 phosphorylation, and further regulated the anti-apoptotic Mcl-1 (84). These results demonstrate that HHT can act as an anti-tumor agent by interfering with the IL-6/JAK1/STAT3 signaling pathway.

### 3.4 Breast cancer

Results from a recent study revealed an increased expression of four anti-apoptotic proteins (Mcl-1, Bcl-2, survivin, XIAP) was observed typically in TNBC cells treated with HHT. There’s a link between the increase in apoptosis in HHT-treated breast cancer cells and the downregulation of the expression of these anti-apoptotic proteins (85). After HHT treatment for 24 hours, chromosomes were shattered, nuclei fragmented, and apoptotic vesicles were generated in MDA-MB-453 cells, indicating that HHT markedly inhibits the proliferation of MDA-MB-453 human breast cancer cells and induces apoptosis in these cells (12). There is increasing evidence showing that microRNAs (miRNAs), a type of small non-coding RNAs, play a crucial role in regulating tumor proliferation and metastasis (86). HHT induces the downregulation of miR-18a-3p and the subsequent inhibition of the AKT/mechanistic Target of Rapamycin (mTOR) pathway, further regulates the expression of Bax/Bcl-2, caspase-3/caspase-9, and poly (ADP-ribose) polymerase (PARP) (87). These downstream molecules are all directly related to apoptosis in breast cancer cells. Plett et al. (88) demonstrated that co-treatment with HHT and paclitaxel is an effective treatment for TNBC cells by enhancing anticancer activity and reducing drug toxicity. Recent studies indicate that HHT dose-dependently reduces the viability of MDA-MB-231 cells, decreases the proportion of CD44^+^/CD24^-^ cells, inhibits tumor sphere formation, and suppresses the expression of Octamer-binding transcription factor 4 (Oct4), CD44, SRY-box transcription factor 2 (Sox2), and Nanog Homeobox (Nanog). Additionally, HHT effectively reduces the stemness, migration, and invasion of triple-negative breast cancer (TNBC) cells by inhibiting the activation of the Hedgehog (HH)/Gli1 Family Zinc Finger 1 (Gli1) signaling pathway (89).

### 3.5 Bladder cancer

Integrins belong to a subfamily of αβ heterodimeric receptors, which embed the cell membrane and regulate tumor proliferation, progression, and metastasis by interacting with cell-cell and cell-matrix complexes (90, 91). Focal adhesion kinase/Src proto-oncogene (FAK/Src) is a hub of integrin-regulated cellular functions that activates the MAPK/ERK and PI3K/AKT signaling pathways (92). HHT significantly inhibits the proliferation of bladder cancer cells and downregulates the expression levels of FAK, Src, ERK, MEK, PI3K, and AKT via the suppression of integrin α5/β1 activity. This suggests that HHT can downregulate the MAPK/ERK and PI3K/AKT signaling pathways and inhibit the progression of tumor metastasis through inhibiting the integrin α5/β1-FAK/Src axis (93). Furthermore, HHT significantly inhibits glycoprotein synthesis in T-24 bladder cancer cells and promotes the accumulation of dolichol-linked oligosaccharides, thereby further suppressing glycosylation (10).

### 3.6 Melanoma

Research indicates that HHT prevents A375 melanoma cells from entering the G2/M phase by regulating the expression of cell cycle-related proteins such as cyclin B1 and CDK1. At the same time, HHT activates the mitochondrial apoptotic signaling pathway, modulating the expression of apoptosis-related proteins, including Bcl-2/Bax, cleaved-caspase 3 and cleaved-PARP, thereby effectively inducing apoptosis (94). In another study, HHT may inhibit the activation of the PI3K/AKT pathway by inhibiting the expression of IRS4 and then downregulating the expression level of cyclin E1/CDK2, thereby blocking the cell cycle in the G0/G1 phase and significantly suppressing the proliferation of A375R cells (95). DNA damage is essential for cell cycle regulation and apoptosis. DNA damage activates the ATM/Checkpoint Kinase 2 (Chk2) signaling pathway, resulting in G2/M phase cell cycle arrest (96). Aurora kinase A (Aurka) and cell division cycle 25c (Cdc25c) are the key regulators of cell division, and Polo-like kinase 1 (Plk1) activates Cdc25c, which mediates G2/M cell cycle progression and promotes mitosis (97). Studies have shown that HHT can cause DNA damage, activate the ATM/Chk2/P53 signaling axis, inhibit the Aurka/Plk1/Cdc25c signaling pathway, and induce G2/M phase cell cycle arrest (98).

### 3.7 Rhabdoid tumors

A rhabdoid tumor (RT) is a type of childhood cancer characterized by a poor prognosis (99). Primary RTs can occur in the brain, kidney, or various soft tissues (100). RT cell lines have been found to be highly sensitive to HHT, and this sensitivity may be associated with low expression of B-cell lymphoma-extra large (BCL-XL), a significant BCL-2 family member involved in apoptotic regulation. Therefore, the low expression of BCL-XL in RT cell lines may make them more sensitive to treatment with HHT (101).

### 3.8 Glioblastoma

Glioblastoma (GBM) is categorized as the primary brain tumor with the highest degree of aggressiveness in adults (102). Platelet-derived growth factor receptor alpha (PDGFRα) is crucial for embryogenesis and organogenesis (103). Activation of PDGFRα initiates multiple signaling pathways, including JAK-STAT (104–106). STAT3 is considered to be one of the hallmarks of GBM aggressiveness and contributes to tumor development and progression in several ways, such as inducing cell proliferation, inhibiting the apoptotic process, and promoting tumor cell migration and invasion (107). HHT treatment of GBM can inactivate STAT3 signaling by inhibiting PDGFRα phosphorylation and PDGFRα/Ras homolog family member A (RhoA)/Rho-associated coiled-coil-containing protein kinase (ROCK) axis to reduce glioblastoma cell proliferation, cytoskeletal remodeling, and migration (108).

**Figure 4:**
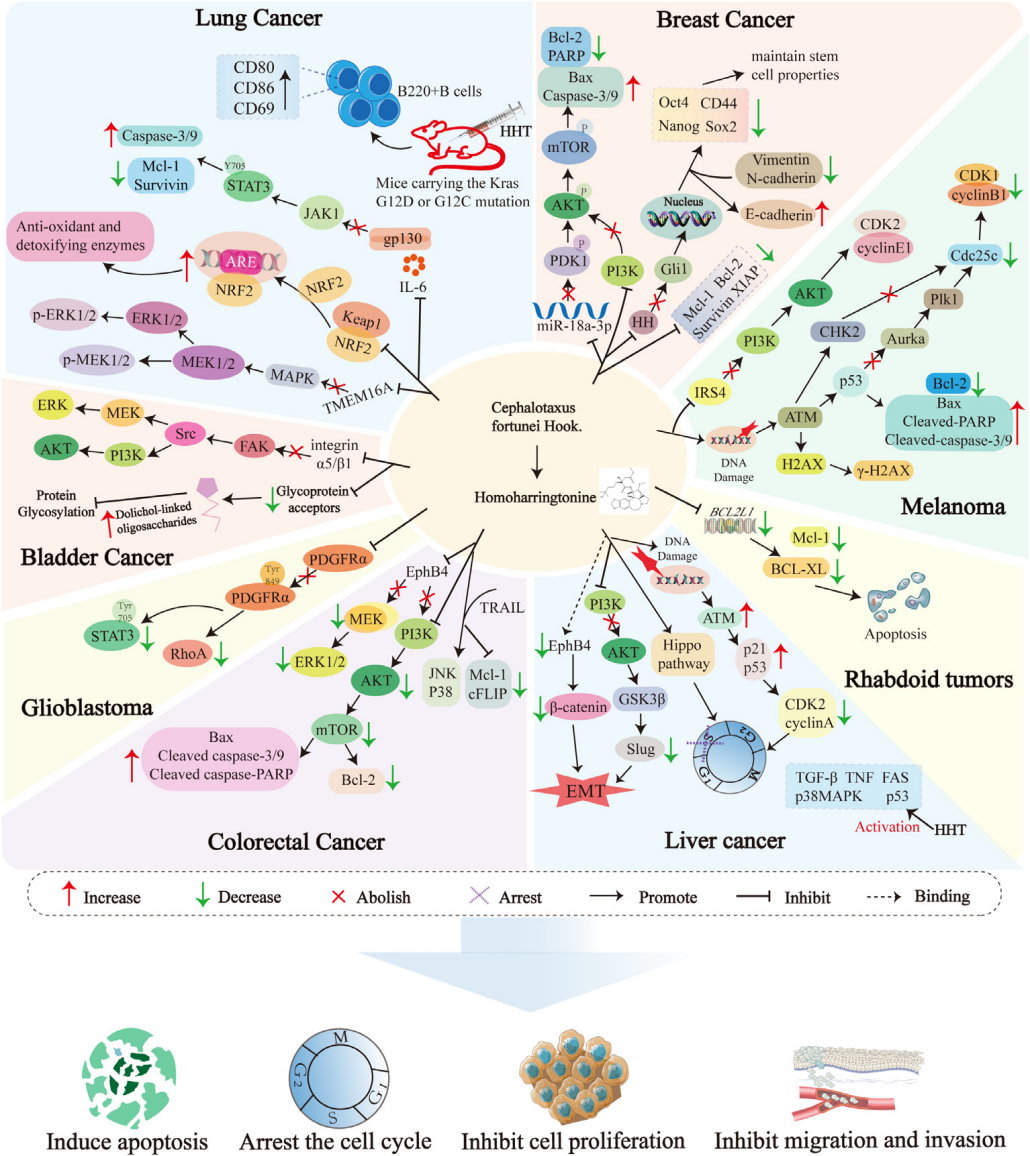
Cell signaling pathways targeted by Homoharringtonine in different diseases. EphB4, Ephrin type-B receptor 4; MAPK, Mitogen-Activated Protein Kinase; ERK1/2, Extracellular Signal-Regulated Kinase 1/2; MEK1/2, Mitogen-Activated Protein Kinase Kinase 1/2; PI3K, Phosphoinositide 3-Kinase; AKT, Protein Kinase B; mTOR, Mechanistic Target of Rapamycin; Bcl-2, B-cell lymphoma-2; Bax, Bcl-2-associated X protein; Mcl-1, Myeloid cell leukemia-1; Caspases, Cysteinyl aspartate-specific protease; TRAIL, TNF-related apoptosis-inducing ligand; cFLIP, Cellular FLICE-inhibitory protein; JNK, c-Jun N-terminal kinase; GSK3β, Glycogen Synthase Kinase 3β; Slug, Snail family transcriptional repressor 2; TGF-β, Transforming growth factor-β; TNF, Tumor Necrosis Factor; FAS, Fas cell surface death receptor; p38MAPK, p38 mitogen-activated protein kinase; FAS, Fas cell surface death receptor; NRF2, Nuclear factor erythroid 2-related factor 2; ARE, Antioxidant Response Element; Keap1, Kelch-like ECH-associated protein 1; TMEM16A, Transmembrane protein 16A; IL-6, Interleukin-6; STAT3, Signal Transducer and Activator of Transcription 3; JAK1, Janus kinase 1; XIAP, X-linked; Inhibitor of Apoptosis Protein; PARP,Poly (ADP-ribose) polymerase; Oct4, Octamer-binding transcription factor 4; Sox2, SRY-box transcription factor 2; Nanog, Nanog Homeobox; PDK1, Pyruvate Dehydrogenase Kinase 1; HH, Hedgehog; Gli1, GLI Family Zinc Finger 1; FAK, Focal adhesion kinase; Src, Src proto-oncogene; ATM, Ataxia-telangiectasia mutated; Chk2, Checkpoint Kinase 2; Aurka, Aurora kinase A; Cdc25c, Cell division cycle 25c; Plk1, Polo-like kinase 1; BCL-XL, B-cell lymphoma-extra large; PDGFRα, Platelet-derived growth factor receptor alpha; RhoA, Ras homolog family member A; H2AX, H2A histone family member X; IRS4, Insulin Receptor Substrate 4; CDK1, Cyclin-Dependent Kinase 1; CDK2, Cyclin-Dependent Kinase 2; EMT, Epithelial-mesenchymal transition.

**Figure 4** illustrates the molecular mechanisms of Homoharringtonine (HHT) in malignant tumors.

## 4 Other pharmacological properties of HHT

### 4.1 Immunomodulatory mechanisms of HHT

Inflammation is a well-recognized hallmark of cancer, and a considerable body of evidence suggests that dysregulation of inflammatory pathways plays a key role in carcinogenesis (109). Inflammatory processes induce elevated levels of pro-inflammatory molecules, such as cytokines (IL-6, IL-2), transcription factors (NF-κB, STAT3), and protein kinase B (AKT), which contribute to cancer initiation and progression (110–112). Studies have shown that HHT exerts its immunomodulatory effects by interacting with a variety of immune mediators, influencing gene transcription, and regulating cellular functions. Key molecules associated with HHT’s immunomodulation will be discussed below. Research has said that HHT affects the activity of certain major transcription factors such as STAT3, NF-κB, and NRF2 which are important in regulating the signaling pathways of pro-inflammatory cytokines and growth factors (113, 114). Moreover, inhibition of STAT3 phosphorylation and nuclear translocation has been demonstrated in HHT-treated cells, which blocks cell growth and metastasis mediated by STAT3 (84, 115). HHT reduces the expression of anti-apoptotic genes regulated by STAT3, such as *Bcl-2* and *Mcl-1*, thereby affecting cell growth and survival in cancer cells (116). NF-κB, an inflammatory transcription factor, is essential for the regulation of a range of genes and biological processes containing immunity, inflammation, cell proliferation and survival (117–121). Previous studies revealed that HHT can inhibit NF-κB activation, which could prevent the inflammatory responses mediated by NF-κB and tumor generation (122). In addition to interfering with transcription factors, HHT also has been shown to have an effect on the production of various cytokines and growth factors. Pro-inflammatory cytokines, such as tumor necrosis factor alpha (TNF-α) and interleukins, have a role in inflammatory processes and growth of neoplastic diseases. HHT has been shown to have an effect on the expression of IL-1, IL-6, and TNF-α. The effect of HHT on downregulating these cytokines affects cell proliferation in general (22).

### 4.2 Antiviral mechanisms of HHT

HHT has been shown to inhibit viral replication in several models of viral infection, impeding protein synthesis and reducing virus production and spread. HHT has also been reported to inhibit coronaviruses, such as SARS-CoV-2, porcine epidemic diarrhea virus (PEDV) (123), and mouse hepatitis virus (MHV) (124), herpesviruses, including varicella-zoster virus (VZV) (125) and herpes simplex virus type 1 (HSV-1) (123), and Rhabdoviruses, such as vesicular stomatitis virus (VSV), pseudorabies virus (PRV), Newcastle disease virus (NDV) (123), and many others.

SARS-CoV-2 is the coronavirus that causes COVID-19. Inhibiting SARS-CoV-2 replication is important for controlling viral infection and disease progression. Studies have demonstrated that HHT can significantly reduce the level of SARS-CoV-2 viral replication (126–130). PEDV-N protein is the nucleocapsid protein of PEDV, which mediates the S-phase host cell block and is crucial for PEDV infection and replication (131). The level of PEDV-N protein in PEDV was significantly reduced after treatment with HHT, effectively reducing the viral load of PEDV-infected cells and animals (123). HHT also successfully inhibits MHV replication in mice by inhibiting viral translocation (124). VZV, a member of the herpesviridae family, is the etiological agent responsible for both varicella (chickenpox) and herpes zoster (shingles) (17). HHT significantly inhibits the mRNA levels of VZV lytic genes, suggesting that HHT may affect the replication and transmission of VZV by inhibiting the expression and replication of VZV lytic genes, in addition to blocking the ribosomal function (125). Eukaryotic translation initiation factor 4E (eIF4E) is a key factor in the initiation of protein synthesis. Phosphorylation of eIF4E regulates the assembly of the eIF4F complex, thereby promoting the translation and replication of certain viruses and affecting the selective specificity of cellular translation (132–135). Dong et al. (123) showed that HHT can antagonize the phosphorylation level of eIF4E in Vero and HeLa cells. Moreover, embryos, chickens, and piglets treated with HHT demonstrated reduced susceptibility to NDV and PEDV infections, indicating that HHT may influence viral replication by inhibiting the phosphorylation of eIF4E. In addition, HHT has been shown to be effective against Hepatitis B, bovine viral diarrhea (136), Ebola (137), and Chikungunya (138) viruses.

### 4.3 Mechanisms of HHT in modulating fibroblast activity and preventing fibrosis

Fibroblast proliferation is considered critical in the formation of fibrotic scar adhesions, while inducing fibroblast apoptosis proves effective in preventing such adhesions (139). The PI3K/AKT/mTOR signaling pathway is pivotal in controlling fibroblast proliferation and apoptosis in mammalian cells (140). Sun et al. (141) have shown that the phosphorylation levels of PI3K, AKT and mTOR in fibroblasts were significantly decreased after HHT treatment for 24 hours. This illustrates that inhibition of the PI3K/AKT/mTOR pathway may be one of mechanisms by which HHT induces apoptosis in fibroblasts and mitigates intra-articular fibrosis after surgery. HHT is also known to inhibit intraocular fibroplasia and vitreous proliferation (142, 143) and may prevent the development of retinal detachment *in vivo (* 144). Similarly, there is evidence that HHT inhibits the proliferation of Human Retro-ocular Fibroblasts (HROF), and the rate of cytostatic inhibition is significantly correlated with HHT concentration (145). In addition, clinical studies have shown that HHT significantly inhibits corneal haze in photorefractive keratectomy (PRK) (146, 147) and is an effective and relatively safe adjunct to glaucoma filtration surgery (148).

### 4.4 Therapeutic mechanisms of HHT in Alzheimer’s disease

Neuroinflammation refers to the inflammatory response occurring within the central nervous system that results from a variety of pathologic insults, including infection, trauma, ischemia, and toxic accumulation (149). Jiang et al. (115) demonstrated that HHT alleviates neuroinflammation in amyloid precursor protein/presenilin 1 (APP/PS1) mice by disrupting the STAT3 pathway, thereby slowing the progression of Alzheimer’s disease.

### 4.5 Mechanisms of HHT in allergic dermatitis treatment

TGF-β inhibits allergic inflammation (150), and HHT exerts anti-inflammatory effects by inducing Ser423/425 phosphorylation of smad3, which activates the TGF-β signaling pathway (18). In addition, HHT inhibits allergic inflammation by regulating the NF-κB/miR-183-5p/B-cell translocation gene 1 (BTG1) axis (122).

## 5 Clinical research and findings on HHT

HHT has demonstrated significant efficacy in the treatment of hematological malignancies, particularly in AML and CML. Currently, the clinical application of HHT is under investigation, including its potential as a monotherapy and in combination with other medications.

HHT has demonstrated significant efficacy in the treatment of AML. A Phase II clinical trial involving 46 patients with refractory and/or relapsed (R/R) AML indicated that 80% of the patients achieved complete remission (151). Despite all patients experiencing severe neutropenia and thrombocytopenia during induction therapy, the therapeutic efficacy of HHT remains noteworthy. Another Phase II clinical trial assessed the efficacy of the combination of sorafenib and HHT in patients with Fms-like tyrosine kinase 3-internal tandem duplication (FLT3-ITD) positive AML (152). Among the 24 patients, 20 of 24 patients (83.3%) achieved complete remission (true or with inadequate hematologic recovery). In addition, in a multicenter Phase II trial, the regimen combining venetoclax, cytarabine, and HHT was found to be promising and well-tolerated for the treatment of R/R AML (153).

In studies of CML, HHT has also demonstrated good clinical efficacy. In 34 patients with CML-chronic myelogenous leukemia in myeloid blast crisis (MBC), HHT in combination with cytarabine (HA regimen) was found to be an effective treatment (154). In 105 patients with Philadelphia chromosome (Ph)-positive CML treated with HHT in combination with low-dose cytarabine, the rate of complete hematologic response during the chronic phase was 72% (155). Additionally, in a study involving 90 patients with Ph-positive early chronic phase CML, a triple therapy regimen consisting of interferon-alpha, cytarabine, and HHT resulted in a complete hematological remission rate of 94%, with 74% of patients achieving cytogenetic remission (156). Although HHT has shown good efficacy in the treatment of CML, it is also associated with certain toxicities. In patients receiving subcutaneous HHT, particularly those who are resistant to imatinib, the tolerance is generally good (157). However, adverse reactions such as bone marrow suppression (151, 158), hypotension (159), mild gastrointestinal toxicity, headaches, and cardiovascular events still occur (155). Therefore, close monitoring of patient responses during clinical application is essential to ensure the safety and efficacy of the treatment.

Furthermore, HHT demonstrates promising potential for the treatment of ocular diseases. Clinical studies indicate that among 36 patients (36 eyes) with globe rupture injuries not associated with retinal detachment who ocular debridement and/or vitrectomy, 18 patients (18 eyes) received injections of HHT. The results showed that HHT significantly inhibited intraocular fibroplasia, reduced the severity of vitreous opacification, and lessened the extent of proliferative vitreoretinopathy. The main side effect observed was transient conjunctival congestion, which typically resolved on its own within 1 to 2 days, with no instances of corneal edema or intraocular hemorrhage noted (143). Additionally, in a study involving 63 patients (73 eyes) with refractory glaucoma, participants were randomly assigned to either the HHT group or the control group, with all patients undergoing the same standard trabeculectomy. Postoperatively, the HHT group received divided conjunctival injections of HHT at a dose of 0.1 mg each. The results showed that the functional 1bleb and success rates of surgery in the HHT group were 73.0% and 75.7%. This further indicates that HHT significantly enhances the long-term efficacy of filtering surgery for refractory glaucoma (148). These findings support the clinical application potential of HHT in the field of ophthalmology.

Although numerous preclinical and early clinical studies support the efficacy of HHT, large-scale, multicenter clinical trials remain scarce, particularly with regard to its application in different types of cancer. Therefore, further clinical trials are needed to validate its efficacy and determine the optimal treatment regimen. Additionally, research on the long-term efficacy and resistance issues of HHT is limited. While its short-term efficacy is notable, the issue of drug resistance during long-term treatment has not been adequately explored and requires further follow-up studies.

Below are some relevant clinical trials on HHT as a therapeutic agent used to treat blood disorders (**Table 1**).

**Table 1:** Homoharringtonine (omacetaxine mepesuccinate) in clinical trials.

Number	Cancer	Title	Trial Phase	Primary Outcomes	References
1	AML	Homoharringtonine in combination with cytarabine for patients with acute myelogenous leukemia	Phase I	CR: Five remissions in 14 patients with relapsed AML. No responses in 8 patients with primary refractory AML. Toxicity: 7Pancytopenia: Universal. Hypotension and fluid retention: More common at higher dose levels. Other mild toxicities: Nausea, vomiting, diarrhea, and mucositis. No significant hepatic, renal, or cardiac toxicity.	(160)
2		Homoharringtonine is safe and effective for patients with acute myelogenous leukemia	Phase II	CR: 7 of 43 patients with relapsed AML achieved CR Response in patients resistant to treatment: 2 of 3 patients resistant to low-dose cytarabine achieved CR.	(161)
3		A phase II study of Homoharringtonine for the treatment of children with refractory or recurrent acute myelogenous leukemia: a pediatric oncology group study	Phase II	CR: Achieved in 4 patients (14% of evaluable patients, 4/28). PR: Achieved in 1 patient (3.6% of evaluable patients, 1/28). ORR: 18% (5/28). MDR: 62 days (range 28-126 days).	(162)
4	AML	Homoharringtonine in combination with cytarabine and aclarubicin in the treatment of refractory/relapsed acute myeloid leukemia: a single-center experience	Phase II	Overall CR rate: 80% of patients achieved complete remission. Single course CR rate: 76.1% after the first course of the HAA regimen. OS: Estimated 3-year OS rate: 42%. RFS: Estimated 3-year RFS rate for 36 CR cases: 49%.	(151)
5		Homoharringtonine (omacetaxine mepesuccinate) as an adjunct for FLT3-ITD acute myeloid leukemia	PhaseII	CHR rate: 83.3% of patients (20/24) achieved complete remission (true or with insufficient hematological recovery). Reduction in ITD Allelic Burden: Significant reduction of FLT3-ITD allelic burden in patients. OS: Median Leukemia-Free Survival: 12 weeks. Median Overall Survival: 33 weeks.	(152)
6		Comparison of efficacy of HCAG and FLAG re-induction chemotherapy in acute myeloid leukemia patients of low- and intermediate-risk groups	Phase II	CR rate: 24% in the HCAG group, 28% in the FLAG group. ORR: 38% in the HCAG group, 42% in the FLAG group. PFS: Median PFS was 29.8 months for the HCAG group, and 30.8 months for the FLAG group. Adverse Effects: Grade 4 Hematological Toxicity: 42 patients in the HCAG group and all patients in the FLAG group. Non-hematological Toxicity: 19 cases in the HCAG group, 40 (78.4%) in the FLAG group.	(163)
7	AML	Sorafenib and omacetaxine mepesuccinate as a safe and effective treatment for acute myeloid leukemia carrying internal tandem duplication of Fms-like tyrosine kinase 3	PhaseII	CR/CRi: Among R/R patients, 28 achieved CR or CRi. Among newly diagnosed patients, 4 achieved CR and 1 achieved CRi. LFS: Median LFS was 5.6 months. OS: Median OS was 10.9 months.	(164)
8		Comparison of efficacy of HCAG and CAG re-induction chemotherapy in elderly low- and intermediate-risk group patients diagnosed with acute myeloid leukemia	Phase II	CR rate: 39.6% in the HCAG group and 33.3% in the CAG group. ORR: 63.0% in the HCAG group and 43.5% in the CAG group PFS: Median PFS was 8.0 months in the HCAG group and 7.0 months in the CAG group.	(165)
9		Optimization of idarubicin and cytarabine induction regimen with homoharringtonine for newly diagnosed acute myeloid leukemia patients based on the peripheral blast clearance rate: A single-arm, phase 2 trial (RJ-AML 2014)	Phase II	CR rate: 84.4% overall, with 87.5% in D5-PBCR (–) group and 80.0% in D5-PBCR (+) group. mOS: Not reached in the entire cohort. mEFS: 42.2 months.	(166)
10	AML	Sorafenib plus triplet therapy with venetoclax, azacitidine and homoharringtonine for refractory/relapsed acute myeloid leukemia with FLT3-ITD: A multicenter phase 2 study	PhaseII	CRc: 76.5% of patients achieved CRc. ORR: 82.4% of patients had a response, including CRc and partial remission. OS: Median OS was 18.1 months. EFS: Median EFS was 11.4 months.	(167)
11		Venetoclax Combined with Azacitidine and Homoharringtonine in Relapsed/Refractory AML: A Multicenter, Phase 2 Trial	PhaseII	CRc: 70.8%, including CR and CRi. MRD Negative: 58.8% of CRc patients achieved MRD-negative status. ORR: 78.1%, including CRc and PR.	(153)
12		Homoharringtonine-based induction regimens for patients with *de-novo* acute myeloid leukamia: a multicenter, open-label, randomized, controlled phase 3 trial	PhaseIII	CR Rate: HAA regimen: 73% (150/206 patients) DA regimen: 61% (125/205 patients) HAD regimen: 67% (133/198 patients) (vs DA, p=0.20) 3-Year EFS: HAA regimen: 35.4% DA regimen: 23.1% HAD regimen: 32.7% (vs DA, p=0.08)	(168)
13	CML	Homoharringtonine and low-dose cytarabine in the management of late chronic-phase chronic myelogenous leukemia	Phase II	CHR Rate: 72% in chronic phase. CRR: 32% (Major response: 15%, Complete response: 5%). Survival: The 4-year survival rate was 55%. Survival was significantly longer with the combination regimen compared to HHT alone after multivariate analysis.	(155)
14		Results of triple therapy with interferon-alpha, cytarabine, and homoharringtonine, and the impact of adding imatinib to the treatment sequence in patients with Philadelphia chromosome-positive chronic myelogenous leukemia in early chronic phase	Phase II	CHR Rate: 94% (85 patients). CRR: 74% (67 patients), with 22% achieving complete response (Ph 0%) and 46% achieving major response (Ph suppression to ≤ 90%). 5-Year Survival Rate: 88%. Blastic Phase Development: Only 9% (8 patients) developed blastic phase.	(156)
15		Phase I/II trial of adding semisynthetic homoharringtonine in chronic myeloid leukemia patients who have achieved partial or complete cytogenetic response on imatinib	Phase I/II	Efficacy: Seven out of 10 evaluable patients showed a decline in BCR-ABL transcript levels. Side Effects: Asthenia: All 10 patients experienced this. Cytopenias: Seen in 3 patients.	(169)
16	CML	Phase I/II study of subcutaneous homoharringtonine in patients with chronic myeloid leukemia who have failed prior therapy	Phase I/II	CHR: Achieved in all 5 evaluable patients. CyR: 3 patients showed cytogenetic responses. BCR-ABL Kinase Domain Mutations: In the 2 patients with BCR-ABL kinase domain mutations at the start, both achieved CG responses, and mutations became undetectable.	(157)
17		A phase II study of continuous infusion homoharringtonine and cytarabine in newly diagnosed patients with chronic myeloid leukemia: CALGB study 19804	Phase II	MCRR: Achieved in 17% of patients (4/23) within nine cycles. CHR rate: 82% of patients (36/44) achieved complete hematologic remission, with the median duration not yet reached.	(158)
18		Prolonged chronic phase in chronic myelogenous leukemia after homoharringtonine therapy	Phase II	CRR: After 12 months of therapy, the cytogenetic response rates were 39/106 for HHT CyR: the rates were 6/18 for HHT Long-term Maintenance of Cytogenetic Response: At the 48-month follow-up, cytogenetic response was maintained in 32/39 patients treated with HHT, indicating long-term efficacy.	(170)
19		Effect of homoharringtonine on bone marrow CD34 + CD117 + cells in patients with chronic myelogenous leukemia	Phase II	Proportion of CD34+ CD117+ cells in bone marrow: Higher in untreated CML patients (24.7%) than in donors (4.4%). Significant decrease in patients who achieved HRR (11.2%) and CRR (8.9%). HRR: Lower in patients with CD34+ CD117+ cells ≥ 20% before treatment (41.7%) compared to those with < 20%.	(171)
20	CML	Subcutaneous omacetaxine mepesuccinate in patients with chronic-phase chronic myeloid leukemia previously treated with 2 or more tyrosine kinase inhibitors including imatinib	Phase II	MCyR: 20% (16 patients), including 8 complete responses. HR: 69% of patients achieved and/or maintained a hematologic response for at least 8 weeks, with a median duration of 12.2 months. Failure-Free Survival: Median of 9.6 months. OS: Median of 34 months. Adverse Events: Common grade 3/4 adverse events included thrombocytopenia (67%), neutropenia (47%), and anemia (37%).	(172)
21		Phase 2 study of subcutaneous omacetaxine mepesuccinate for chronic-phase chronic myeloid leukemia patients resistant to or intolerant of tyrosine kinase inhibitors	Phase II	HR: 67% of patients achieved or maintained a hematologic response; the median duration of response was 7.0 months. MCyR: 22% of patients achieved MCyR, including 4% with complete cytogenetic responses. PFS: Median of 7.0 months. OS: Median of 30.1 months.	(173)
22		Phase 2 study of subcutaneous omacetaxine mepesuccinate after TKI failure in patients with chronic-phase CML with T315I mutation	Phase II	CHR: Achieved in 48 patients (77%; 95% lower confidence limit, 65%); median response duration was 9.1 months. MCyR: 23% of patients achieved MCyR, including 16% with complete cytogenetic response. PFS: Median of 7.7 months.	(174)
23	CML	Omacetaxine mepesuccinate in patients with advanced chronic myeloid leukemia with resistance or intolerance to tyrosine kinase inhibitors	PhaseII	MHR: 37% in patients with accelerated phase chronic myeloid leukemia (AP-CML). 9% in patients with myeloid blast phase chronic myeloid leukemia (BP-CML). MHR in patients with a history of T315I mutation: 22% in AP-CML and 5% in BP-CML.	(175)
24		Homoharringtonine combined with cytarabine to treat chronic myelogenous leukemia in myeloid blast crisis and its impact on bone marrow CD34+CD7+ cells	Phase II	OHR: 60.1% (complete/partial hematological response and hematological improvement). CyR: 21.2% of patients achieved a cytogenetic response 12 months after treatment. Change in Bone Marrow CD34+CD7+ Cells: The proportion of CD34+CD7+ cells in bone marrow decreased from 19.4 ± 7.9% (before treatment) to 14.1 ± 7.1% after treatment (p < 0.05).	(154)
25	CMML MDS	A phase II study of omacetaxine mepesuccinate for patients with higher-risk myelodysplastic syndrome and chronic myelomonocytic leukemia after failure of hypomethylating agents	Phase II	ORR: 33% OS: Median OS of 7.5 months; 1-year OS rate was 25%.	(176)
26	MDS	Homoharringtonine in patients with myelodysplastic syndrome (MDS) and MDS evolving to acute myeloid leukemia	Phase II	ORR: 28% (8/28) CR: Achieved in 7 patients. Myelosuppression: 13 induction-related deaths due to neutropenic infections.	(177)
27		A phase II open-label study of the intravenous administration of homoharringtonine in the treatment of myelodysplastic syndrome	Phase II	CHR and CyR: One patient (11%) after one course of HHT treatment. Grade 3/4 Myelosuppression: Observed in 56% (5/9) of patients.	(178)
28		The efficacy and toxicity of the CHG priming regimen (low-dose cytarabine, homoharringtonine, and G-CSF) in higher risk MDS patients relapsed or refractory to decitabine	Phase II	ORR: 39.4% (13 out of 33 patients). CR rate: 27.3% (9 out of 33 patients). mCRR: 6.1% (2 out of 33 patients). PR rate: 6.1% (2 out of 33 patients). OS: Median OS of 7.0 months for the 33 patients.	(160)
29	MDS t-AML	Effect of low-dose cytarabine, homoharringtonine and granulocyte colony-stimulating factor priming regimen on patients with advanced myelodysplastic syndrome or acute myeloid leukemia transformed from myelodysplastic syndrome	Phase II	ORR: 71.9% CR rate: 46.9% (15 patients). PR rate: 25% (8 patients). mOS: 18.2 months. Duration of CR: 10.6 months for patients who received alternative chemotherapy.	(179)

AML, Acute Myeloid Leukemia; CML, Chronic Myeloid Leukemia; MDS, Myelodysplastic Syndromes; t-AML, Transformed Acute Myeloid Leukemia; CR, Complete Remission; PR, Partial Remission; ORR, Overall Response Rate; MDR, Median Duration of Response; RFS, Relapse-Free Survival; OS, Overall Survival; CHR, Complete Hematologic Remission; PFS, Progression-Free Survival; CR/CRi, Composite Complete Remission; LFS, Leukemia-Free Survival; mOS, Median Overall Survival; mEFS, Median Event-Free Survival; CRc, Composite Complete Remission Rate; EFS, Event-Free Survival; MRD, Measurable Residual Disease; CRR, Cytogenetic Response Rate; CyR, Cytogenetic Response; MCRR, Major Cytogenetic Response Rate; HRR, Hematological remission rate; MCyR, Major Cytogenetic Response; HR, Hematologic Response; MHR, Major Hematologic Response; OHR, Overall Hematological Respons

## 6 Current challenges in the clinical application of HHT

Despite its promising potential in treating various malignancies and other diseases, the clinical application of HHT faces several challenges. HHT is primarily used for the treatment of hematological tumors, but it is also associated with certain side effects, including bone marrow suppression, gastrointestinal adverse reactions (such as nausea, vomiting, and diarrhea), as well as potential cardiotoxicity, hypotension, and hyperglycemia (23). The adverse effects and toxicities of HHT are related to its therapeutic effects and are due to the drug’s impact on normal cells and tissues. Bone marrow suppression is one of the most common adverse effects and can lead to a reduction in the counts of leukocytes, erythrocytes, and platelets (174),increasing the risk of infection, anemia, and bleeding. Cardiotoxicity may manifest as arrhythmias and myocardial damage (180). Adverse reactions from treatment with HHT can include gastrointestinal symptoms of nausea, vomiting, and dyspepsia, which might be connected to the drug irritating the gastrointestinal tract directly or causing a change in the permeability of the barrier of the intestinal epithelial cell, and hypotension and hyperglycemia can also develop due to vascular and metabolic influence of the drug (181, 182). Moreover, HHT may lead to ocular responses during the ocular disease therapy, such as vessel dilation of ocular, hyperemia of conjunctival, intraocular bleeding, or edema of corneal, but these ocular responses tend to be transient and last for a week (143). Overall, HHT is a potent chemotherapeutic agent with significant therapeutic effects, but close attention must be paid to possible adverse reactions and toxic side effects during its use.

Furthermore, the poor solubility of HHT limits its bioavailability and effectiveness in clinical applications. To address this, researchers are actively exploring innovative drug delivery systems, such as nanocarriers and liposomes, to enhance HHT’s solubility and targeted delivery capabilities, thereby improving its therapeutic efficacy (24, 183). Currently, there are relatively few large-scale clinical trials assessing the efficacy and safety of HHT, making it increasingly urgent to establish standardized treatment protocols for different patient populations. Therefore, conducting more comprehensive studies will help deepen our understanding of HHT’s clinical application potential.

## 7 Strategies to enhance the bioavailability of HHT

The clinical application of HHT remains constrained by its limited solubility. Research has demonstrated that HHT exhibits a synergistic effect when used in conjunction with other therapeutic agents, thereby enhancing its anti-tumor efficacy. To address the solubility challenge and improve the clinical utility of HHT, scientists are investigating multiple strategies. These include combining HHT with other drugs, developing novel delivery methods, adopting advanced drug delivery systems, and optimizing the molecular structure to enhance efficacy. Such innovative approaches hold promise for improving the clinical performance of HHT while mitigating its potential toxicity.

### 7.1 Targeted delivery systems to maximize HHT therapeutic potential

Several drug delivery systems have been developed to address the distinctive characteristics of HHT in terms of low solubility and to improve solubilization, stabilization, and targetability to optimize pharmacological activities. A range of drug delivery systems, including nanoparticles, polymeric encapsulation and liposomes, have been employed for HHT delivery. By encapsulating HHT within a delivery vehicle, HHT solubilization, stability, pharmacokinetics, and bioavailability have been improved, and targeted delivery to tumors has been realized.

Among them, a co-delivery system of homoharringtonine and doxorubicin (183),high proportion PEG of long-circulating HHT liposomes (LCL-HHT-H-PEG) (184),and magnetic Fe_3_O_4_ nanoparticles (HHT-MNP-Fe_3_O_4_) (185) all promoted apoptosis, inhibited cell proliferation, and exhibited significant cytotoxicity. Long-circulating PEGylated liposomes loaded with HHT (LC-Lipo-HHT) have good biocompatibility and a high safety profile, reducing HHT irritation in the vasculature (186).Compared to DNR/HHT co-delivery liposomes without folic acid modification (DH-LP), folic acid-modified DNR and HHT concomitantly transmitted liposomes (FA-DH-LP) have stronger cell toxicity and a better capacity to target tumors (187). In addition, HHT-loaded PLGA-SS-PEG nanodrugs demonstrate enhanced therapeutic efficacy and reduced toxicity (24). The application of these nanoparticle drugs significantly improves the intracellular uptake efficiency and efficacy of therapeutic agents while reducing side effects.

### 7.2 Combining HHT with other medicines

A variety of combination drug delivery regimens and delivery systems are used to treat hematologic diseases, which may improve drug absorption, cytotoxicity, and safety compared to drug administration alone or in combination with drugs. The combination of HHT and other drugs, such as imatinib, bortezomib, and apatinib, can lead to a synergistic effect, which would enhance their anticancer effects against leukemia. These regimens with HHT have multi-target and multi-pathways acting on leukemia cells, which can increase the therapeutic effect of the drugs and reduce the occurrence of drug resistance.

**Figure 5:**
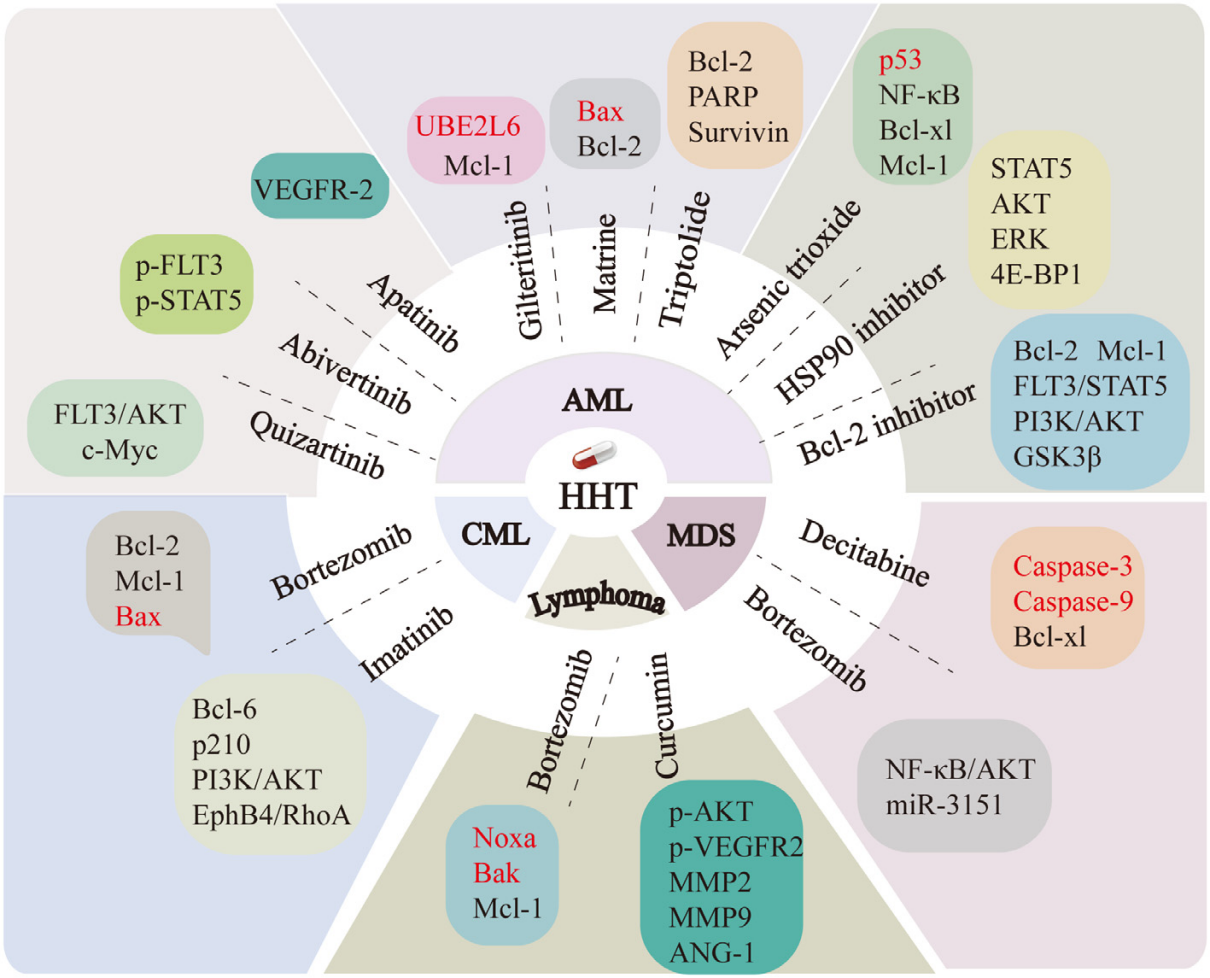
The combination of HHT with other drugs enhances therapeutic efficacy by regulating the expression of multiple genes. Targets downregulated by HHT are indicated in black, while those upregulated are marked in red. PI3K, Phosphoinositide 3-Kinase; AKT, Protein Kinase B; c-Myc, Cellular Myc; p-STAT5, Phosphorylated Signal Transducer and Activator of Transcription 5; p-FLT3, Phosphorylated FMS-like tyrosine kinase 3; VEGFR-2, Vascular Endothelial Growth Factor Receptor 2; UBE2L6, Ubiquitin Conjugating Enzyme E2 L6; Mcl-1, Myeloid cell leukemia-1; Bax, Bcl-2-associated X protein; Bcl-2, B-cell lymphoma-2; PARP,Poly (ADP-ribose) polymerase; NF-κB, Nuclear factor kappa B; BCL-XL, B-cell lymphoma-extra large; ERK, Extracellular Signal-Regulated Kinase; 4E-BP1, eIF4E-Binding Protein 1; GSK3β, Glycogen Synthase Kinase 3β; Caspases3/9, Cysteinyl aspartate-specific protease3/9; MMP2/9, Matrix Metalloproteinase2/9; ANG-1, Angiopoietin-1; Noxa, NADPH oxidase activator; Bak, Bcl-2 homologous antagonist/killer; BCL-6, B-cell lymphoma 6; EphB4, Ephrin type-B receptor 4; RhoA, Ras homolog gene family member A.

HHT and bortezomib (BTZ) are two commonly used anticancer drugs that show potential for synergistic effects in the management of leukemia and other malignancies. In the cells cultured *in vitro*, the synergistic use of HHT and BTZ enhanced the cytotoxicity of BTZ against K562 leukemia cells, reduced the expression of the anti-apoptotic proteins Bcl-2 and Mcl-1, while enhancing the expression of the pro-apoptotic protein Bax (15). In DLBCL and mantle cell lymphoma (MCL) cells, HHT and BTZ synergistically enhanced the expression levels of pro-apoptotic proteins Noxa and Bak, while concurrently downregulating the expression of the anti-apoptotic protein Mcl-1 (19). The above results suggest that HHT in combination with BTZ can affect cell growth by regulating apoptosis-related proteins. Additionally, Zhang et al. (14) reported that the combination of HHT and BTZ influenced the proliferation of SKM-1 cells, a myelodysplastic syndrome (MDS) cell line, by interfering with the AKT and NF-κB signaling pathways, upregulating the expression of their downstream oncogene p53, and decreasing the expression of miR-3151. In addition, the combination of HHT and decitabine (DAC) induced apoptosis in the MDS cell line SKM-1 by upregulating the expression of pro-apoptotic proteins caspase-3 and caspase-9, while downregulating the expression of the anti-apoptotic protein BCL-XL (30). Co-treatment with HHT and imatinib (IM) significantly suppressed proliferation and induced apoptosis in CML K562 cells. A prior study showed that HHT and IM synergistically inhibited the expression level of Zinc finger X-linked protein and interfered with the PI3K/AKT pathway (188), Bcl-6 expression (189), and p210 protein expression and its kinase activity (190). Co-treatment with HHT and IM also augmented the sensitivity of K562 cells to IM and the anti-tumor activity of imatinib by blocking the EphB4/RhoA pathway (191). Furthermore, BCR-ABL is a critical oncogene that mediates the malignant proliferation observed in hematopoietic stem and progenitor cells in CML (192). HHT induces autophagy and promotes ubiquitination of BCR-ABL in CML cells. The ubiquitinated BCR-ABL binds to p62, facilitating the degradation of the BCR-ABL protein and inducing apoptosis in imatinib-resistant CML K562G cells (193). FLT3-ITD leads to constitutive activation and autophosphorylation of FLT3, triggers the activation of various intracellular signaling pathways, promotes independent cell proliferation, and is crucial in the development and progression of AML (194, 195). Specific inhibition of FLT3 kinase activity represents a crucial strategy in the treatment of AML. HHT can be administered in conjunction with other therapeutic agents to treat leukemia associated with FLT3-ITD mutations by modulating cell signaling pathways, inducing apoptosis, and affecting stem cell properties. HHT in combination with quizartinib has been documented to exhibit anti-leukemic properties through the modulation of the FLT3-AKT-c-Myc signaling pathway and the reduction of side population and aldehyde dehydrogenase-positive cells possessing leukemic stem cell properties (196). HHT in combination with abivertinib affects leukemia cell growth by targeting the phosphorylation of the BTK and PI3K pathways and downregulating the expression of p-FLT3 and p-STAT5 (197). HHT in combination with heat shock protein 90 (HSP90) inhibitors synergistically reduces FLT3 expression and inhibits downstream signaling pathways, including STAT5, AKT, ERK, and 4E-BP1, demonstrating efficacy in treating FLT3-ITD-positive acute myeloid leukemia (AML) (198). The combination treatment of HHT and apatinib can exert its anti-leukemic effects by inhibiting the VEGFR-2 signaling pathway (199). HHT and gilteritinib can upregulate Ubiquitin Conjugating Enzyme E2 L6 (UBE2L6) together, promoting the degradation of Mcl-1 through the ubiquitin-proteasome pathway, and bring an effective drug target for FLT3-ITD mutated leukemia (200). Bcl-2 is a potent anti-apoptotic protein pivotal in mediating resistance to chemotherapy (201, 202). HHT combined with a Bcl-2 inhibitor antagonizes the FLT3-STAT5 pathway by downregulating the expression of Bcl-2 and Mcl-1 (203) and also interferes with the PI3K/AKT/GSK3β pathway to effectively treat leukemia (204). AML1-ETO is one of the hallmark features of t (7, 20) AML. Given its pivotal role in the pathogenesis of AML, especially in the M2 subtype, inhibiting AML1-ETO has become an important therapeutic strategy for treating AML (205). Further studies have demonstrated that the combination of HHT with aclarubicin and cytarabine significantly induces apoptosis in t (7, 20) type AML cells. This effect is mediated through caspase-3-dependent cleavage of AML1-ETO, thereby exerting an antitumor effect (206). Evidence suggests that HHT also has anti-leukemic effects when combined with oridonin (207), triptolide (208), arsenic trioxide (209–211), matrine (212), and AG490 (a JAK2 inhibitor) (213). In addition, HHT combined with curcumin significantly suppresses the proliferation, migration, and angiogenesis of lymphoma cells by inhibiting the phosphorylation of VEGFR2 and AKT, which reduces the signaling of angiogenin 1 (ANG-1), matrix metalloproteinase 2 (MMP2), and matrix metalloproteinase 9 (MMP9) (29). The mechanisms by which HHT synergizes with other drugs are illustrated in **Figure 5**.

### 7.3 Development and optimization of homoharringtonine derivatives

With the in-depth investigation into the anti-cancer mechanisms of HHT, researchers have refined its molecular structure to enhance antitumor efficacy and mitigate toxicity, particularly in the treatment of CML and other myeloid malignancies (7, 13, 214). Clinical studies have shown that semi-synthetic homoharringtonine (sHHT) significantly reduces BCR-ABL transcript levels, particularly in Philadelphia chromosome-positive (Ph+) CML patients with poor response to imatinib, with excellent tolerability (169). Additionally, semi-synthetic homoharringtonine (ssHHT) demonstrates favorable pharmacokinetics and low inter-patient variability in patients with advanced AML (215). Furthermore, BS-HH-002, a novel HHT derivative, has demonstrated significantly enhanced anti-tumor activity through structural optimization. Specifically, in the treatment of pancreatic cancer, it effectively inhibits cell proliferation and induces apoptosis by degrading the anti-apoptotic protein MCL-1. Additionally, BS-HH-002 exhibits superior pharmacokinetic properties and circumvents the cardiotoxicity issues associated with traditional HHT (216). With the ongoing refinement of semi-synthetic HHT and its derivatives, coupled with the advancement of clinical research, the potential of HHT in treating various tumors is anticipated to be more extensively explored. Moreover, by developing innovative drug delivery routes and systems, it is possible to further address the solubility limitations of traditional HHT, thereby enhancing its bioavailability.

## 8 Conclusions and future perspectives

Plant-derived HHT is a multi-pathway and multi-target chemotherapeutic agent that demonstrates extensive potential for the treatment of various diseases. By inhibiting protein synthesis and modulating critical cell signaling pathways such as PI3K/AKT, FAK/Src, and MAPK/ERK, HHT regulates the expression of cyclins and several genes associated with cell survival and apoptosis, including members of the caspase family, Bcl-2 family, Mcl-1, Noxa, Bad, survivin, and XIAP. Consequently, HHT exerts its pharmacological effects, which include anti-tumor, anti-viral, and anti-fibrotic activities. While HHT has shown significant clinical efficacy in hematological malignancies such as CML and AML, side effects including myelosuppression and cardiotoxicity restrict its long-term application. Consequently, research into drug combinations and drug delivery systems offers promising avenues for optimizing treatment protocols. When combined with other agents (e.g., imatinib, bortezomib, and apatinib), HHT can operate via multiple mechanisms, such as disrupting the PI3K/AKT pathway, modulating apoptosis-related proteins, and diminishing the population of cells with leukemia stem cell characteristics, thereby inhibiting leukemia cell proliferation, enhancing therapeutic efficacy, and mitigating the development of drug resistance. Furthermore, the limited bioavailability of HHT constrains its broad application. Current research efforts are concentrated on enhancing its bioavailability via improved drug delivery systems. These systems, such as nanoparticles, polymer encapsulation, and liposomes, can significantly improve the water solubility and stability of HHT, thereby increasing its *in vivo* absorption rate. Such enhancements not only augment the therapeutic efficacy of HHT but also effectively mitigate its toxic side effects. Recent studies have also uncovered the antiviral potential of HHT, especially in the management of chronic viral infections like hepatitis B.

While HHT has shown promising short-term efficacy in clinical studies, long-term efficacy and drug resistance remain significant challenges for ongoing research. Future investigations should delve deeper into the molecular mechanisms of HHT, particularly its regulatory functions in various signaling pathways, to further elucidate its potential mechanisms in anticancer, antiviral, and anti-fibrosis applications.

Additionally, the development of more efficient drug delivery systems could enhance the targeting and bioavailability of HHT while mitigating toxic side effects. In terms of clinical application, research on the synergistic use of HHT with other therapeutic agents, such as targeted therapies and immunotherapies, should be intensified to explore its advantages in combination treatments. In addition to its established role in hematological malignancies, the potential applications of HHT in solid tumors, viral infections, fibrosis, and neurodegenerative diseases warrant further investigation. While HHT has been extensively studied in hematological cancers, research on its efficacy in other malignant and non-malignant conditions remains limited. Future studies should aim to broaden the scope of HHT’s therapeutic applications in these areas. Moreover, addressing the management of HHT-related side effects and toxicity continues to be a critical challenge in clinical practice, which merits further exploration in upcoming research.

In summary, HHT, a promising anticancer agent, has the potential for substantial health benefits. However, drug resistance and adverse effects remain challenges in long-term administration. Future research should focus on large-scale, multicenter clinical trials to optimize drug delivery systems for HHT, improve its bioavailability, overcome drug resistance, and explore its potential applications in fields such as immunotherapy and antiviral therapy.

## References

[1] Smith CR Jr Powell RG Mikolajczak KL. The genus Cephalotaxus: source of homoharringtonine and related anticancer alkaloids. Cancer Treat Rep. (1976) 60:1157–70. 791485

[2] Man S Gao W Wei C Liu C. Anticancer drugs from traditional toxic Chinese medicines. Phytother Res. (2012) 26:1449–65. doi: 10.1002/ptr.4609 22389143

[3] Hao DC Hou XD Gu XJ Xiao PG Ge GB. Ethnopharmacology, chemodiversity, and bioactivity of Cephalotaxus medicinal plants. Chin J Nat Med. (2021) 19:321–38. doi: 10.1016/S1875-5364(21)60032-8 33941338

[4] Zhongzhen G. Clinical research on alkaloids from cephalotaxus plants. Cancer Res Prev Treat. (1976) 2):20–8.

[5] Zhongwu M. Cephalotaxus: A novel medicine for treating leukemia. Life World. (1978) 03):35–6.

[6] Powell RG Weisleder D Smith CR Wolff IA. Structure of cephalotaxine and related alkaloids. Tetrahedron Lett. (1969) 10:4081–4. doi: 10.1016/S0040-4039(01)88620-2

[7] Powell RG Weisleder D Smith CR Rohwedder WK. Structures of harringtonine, isoharringtonine, and homoharringtonine. Tetrahedron Lett. (1970) 11:815–8. doi: 10.1016/S0040-4039(01)97839-6 5436615

[8] Powell RG Weisleder D Smith CR. Antitumor alkaloids from cephalotaxus harringtonia: structure and activity. J Of Pharm Sci. (1972) 61:1227–30. doi: 10.1002/jps.2600610812 5050371

[9] Luo M Bian S Xue Y. Study on combined chemosensitivity test in acute non-lymphocytic leukemia. Zhonghua Xue Ye Xue Za Zhi. (1998) 19:59–62. 10921102

[10] Ling YH Tseng MT Harty JI. Effects of homoharringtonine on protein glycosylation in human bladder carcinoma cell T-24. Cancer Res. (1989) 49:76–80. 2908854

[11] Jin W Qu LF Chen Q Chang XZ Wu J Shao ZM. Gene expression pattern in apoptotic QGY-7703 cells induced by homoharringtonine. Acta Pharmacol Sin. (2007) 28:859–68. doi: 10.1111/j.1745-7254.2007.00569.x 17506945

[12] Lei R Nali C Shuyin L Bei P. A study of homoharringtonine in anti-breast cancer. Med Pharm J Chin People’s Liberation Army. (2009) 21:19–21 + 94. doi: 10.3969/j.issn.2095-140X.2009.01.008

[13] Kantarjian HM O’Brien S Cortes J. Homoharringtonine/omacetaxine mepesuccinate: the long and winding road to food and drug administration approval. Clin Lymphoma Myeloma Leukemia. (2013) 13:530–3. doi: 10.1016/j.clml.2013.03.017 PMC3775965 23790799

[14] Zhang J Chen B Wu T Wang Q Zhuang L Zhu C. Synergistic effect and molecular mechanism of homoharringtonine and bortezomib on SKM-1 cell apoptosis. PloS One. (2015) 10:e0142422. doi: 10.1371/journal.pone.0142422 26544558 PMC4636319

[15] Xie Chun TA. Combined effect of bortezomib and homoharringtonine on K562 cells and their mechanisms. J Exp Hematol. (2018) 26:395–400. doi: 10.7534/j.issn.1009-2137.2018.02.014 29665904

[16] Tujebajeva RM Graifer DM Karpova GG Ajtkhozhina NA. Alkaloid homoharringtonine inhibits polypeptide chain elongation on human ribosomes on the step of peptide bond formation. FEBS Lett. (1989) 257:254–6. doi: 10.1016/0014-5793(89)81546-7 2583270

[17] Arvin AM. Varicella-zoster virus. Clin Microbiol Rev. (1996) 9:361–81. doi: 10.1128/CMR.9.3.361 PMC172899 8809466

[18] Chen J Mu Q Li X Yin X Yu M Jin J. Homoharringtonine targets Smad3 and TGF-β pathway to inhibit the proliferation of acute myeloid leukemia cells. Oncotarget. (2017) 8:40318–26. doi: 10.18632/oncotarget.16956 PMC5522237 28454099

[19] Nguyen T Parker R Zhang Y Hawkins E Kmieciak M Craun W. Homoharringtonine interacts synergistically with bortezomib in NHL cells through MCL-1 and NOXA-dependent mechanisms. BMC Cancer. (2018) 18:1129. doi: 10.1186/s12885-018-5018-x 30445933 PMC6240231

[20] Shi X Zhu M Gong Z Yang T Yu R Wang J. Homoharringtonine suppresses LoVo cell growth by inhibiting EphB4 and the PI3K/AKT and MAPK/EKR1/2 signaling pathways. Food Chem Toxicol. (2020) 136:110960. doi: 10.1016/j.fct.2019.110960 31726078

[21] Qu M Li J Yuan L. Uncovering the action mechanism of homoharringtonine against colorectal cancer by using network pharmacology and experimental evaluation. Bioengineered. (2021) 12:12940–53. doi: 10.1080/21655979.2021.2012626 PMC8810123 34847838

[22] Liu J Shi L Huang W Zheng Z Huang X Su Y. Homoharringtonine attenuates dextran sulfate sodium-induced colitis by inhibiting NF-κB signaling. Mediators Inflammation. (2022) 2022:3441357. doi: 10.1155/2022/3441357 PMC9536985 36211988

[23] Kantarjian HM Talpaz M Santini V Murgo A Cheson B O’Brien SM. Homoharringtonine: history, current research, and future direction. Cancer. (2001) 92:1591–605. doi: 10.1002/1097-0142(20010915)92:6&lt;1591::aid-cncr1485<3.0.co;2-u 11745238

[24] Zhang Z Cheng W Pan Y Jia L. An anticancer agent-loaded PLGA nanomedicine with glutathione-response and targeted delivery for the treatment of lung cancer. J Mater Chem B. (2020) 8:655–65. doi: 10.1039/c9tb02284h 31904073

[25] Short NJ Rytting ME Cortes JE. Acute myeloid leukaemia. Lancet. (2018) 392:593–606. doi: 10.1016/S0140-6736(18)31041-9 30078459 PMC10230947

[26] Chen XJ Zhang WN Chen B Xi WD Lu Y Huang JY. Homoharringtonine deregulates MYC transcriptional expression by directly binding NF-κB repressing factor. Proc Natl Acad Sci U.S.A. (2019) 116:2220–5. doi: 10.1073/pnas.1818539116 PMC6369765 30659143

[27] Jin C Minran Z Ting S Xuemei Q Zhongmin C Chunyan C. Inhibition of FoxM1 sensitizes leukemia K562 cells to homoharringtonine. Chin J Pathophysiology. (2015) 31:1928–32. doi: 10.3969/j.issn.1000-4718.2015.11.002

[28] Li C Dong L Su R Bi Y Qing Y Deng X. Homoharringtonine exhibits potent anti-tumor effect and modulates DNA epigenome in acute myeloid leukemia by targeting SP1/TET1/5hmC. Haematologica. (2020) 105:148–60. doi: 10.3324/haematol.2018.208835 PMC6939512 30975912

[29] Zhang Y Xiang J Zhu N Ge H Sheng X Deng S. Curcumin in combination with omacetaxine suppress lymphoma cell growth, migration, invasion, and angiogenesis via inhibition of VEGF/akt signaling pathway. Front Oncol. (2021) 11:656045. doi: 10.3389/fonc.2021.656045 34458134 PMC8386016

[30] Geng S Yao H Weng J Tong J Huang X Wu P. Effects of the combination of decitabine and homoharringtonine in SKM-1 and Kg-1a cells. Leuk Res. (2016) 44:17–24. doi: 10.1016/j.leukres.2016.02.002 26991610

[31] Xie WZ Lin MF Huang H Cai Z. Homoharringtonine-induced apoptosis of human leukemia HL-60 cells is associated with down-regulation of telomerase. Am J Chin Med. (2006) 34:233–44. doi: 10.1142/S0192415X06003795 16552835

[32] Zhang J Gan Y Li H Yin J He X Lin L. Inhibition of the CDK2 and Cyclin A complex leads to autophagic degradation of CDK2 in cancer cells. Nat Commun. (2022) 13:2835. doi: 10.1038/s41467-022-30264-0 35595767 PMC9122913

[33] Zhang T Shen S Zhu Z Lu S Yin X Zheng J. Homoharringtonine binds to and increases myosin-9 in myeloid leukaemia. Br J Pharmacol. (2016) 173:212–21. doi: 10.1111/bph.13359 PMC4813388 26448459

[34] Huang MT. Harringtonine, an inhibitor of initiation of protein biosynthesis. Mol Pharmacol. (1975) 11:511–9. 1237080

[35] Fresno M Jiménez A Vázquez D. Inhibition of translation in eukaryotic systems by harringtonine. Eur J Biochem. (1977) 72:323–30. doi: 10.1111/j.1432-1033.1977.tb11256.x 319998

[36] Gandhi V Plunkett W Cortes JE. Omacetaxine: a protein translation inhibitor for treatment of chronic myelogenous leukemia. Clin Cancer Res. (2014) 20:1735–40. doi: 10.1158/1078-0432.CCR-13-1283 PMC4048124 24501394

[37] Nourbakhsh M Oumard A Schwarzer M Hauser H. NRF, a nuclear inhibitor of NF-kappaB proteins silencing interferon-beta promoter. Eur Cytokine Netw. (2000) 11:500–1. 11203193

[38] Kalin TV Ustiyan V Kalinichenko VV. Multiple faces of FoxM1 transcription factor: lessons from transgenic mouse models. Cell Cycle. (2011) 10:396–405. doi: 10.4161/cc.10.3.14709 21270518 PMC3115014

[39] Pugh BF Tjian R. Mechanism of transcriptional activation by Sp1: evidence for coactivators. Cell. (1990) 61:1187–97. doi: 10.1016/0092-8674(90)90683-6 2194667

[40] Suske G. The Sp-family of transcription factors. Gene. (1999) 238:291–300. doi: 10.1016/s0378-1119(99)00357-1 10570957

[41] Bray JK Dawlaty MM Verma A Maitra A. Roles and regulations of TET enzymes in solid tumors. Trends Cancer. (2021) 7:635–46. doi: 10.1016/j.trecan.2020.12.011 33468438

[42] Robertson D. Cancer researchers validate and pursue telomerase. Nat Biotechnol. (1997) 15:518. doi: 10.1038/nbt0697-518 9181571

[43] Gorbunova V Seluanov A. Telomerase as a growth-promoting factor. Cell Cycle. (2003) 2:534–7. doi: 10.4161/cc.2.6.515 14504469

[44] Ohyashiki JH Ohyashiki K Iwama H Hayashi S Toyama K Shay JW. Clinical implications of telomerase activity levels in acute leukemia. Clin Cancer Res. (1997) 3:619–25. 9815729

[45] Engelhardt M Mackenzie K Drullinsky P Silver RT Moore MA. Telomerase activity and telomere length in acute and chronic leukemia, pre- and post-ex vivo culture. Cancer Res. (2000) 60:610–7. 10676644

[46] Yihan Wuj D Qian Dz W Yufeng LI. Mechanism of mTOR Pathway in K562 cell Apoptosis Induced by Homoharringtonine. J Exp Hematol. (2018) 26:105–9. doi: 10.7534/j.issn.1009-2137.2018.01.017 29397826

[47] Xue-Ying Yel L Yu-Ye Dy S Llyu-F. Effect of autophagy on homoharringtonine-treated K562 cells. J Exp Hematol. (2017) 25:412–7. doi: 10.7534/j.issn.1009-2137.2017.02.019 28446285

[48] Chen P Yuan Q Yang H Wen X You P Hou D. Homoharringtonine enhances bortezomib antimyeloma activity in myeloma cells adhesion to bone marrow stromal cells and in SCID mouse xenografts. Leuk Res. (2017) 57:119–26. doi: 10.1016/j.leukres.2017.04.007 28463768

[49] Zhang Y Huang N Xu J Zheng W Cui X. Homoharringtonine exerts an antimyeloma effect by promoting excess parkin-dependent mitophagy. Drug Des Devel Ther. (2020) 14:4749–63. doi: 10.2147/DDDT.S279054 PMC7652225 33177810

[50] Alaggio R Amador C Anagnostopoulos I Attygalle AD Araujo I Berti E. The 5th edition of the world health organization classification of haematolymphoid tumours: lymphoid neoplasms. Leukemia. (2022) 36:1720–48. doi: 10.1038/s41375-022-01620-2 PMC9214472 35732829

[51] Klanova M Andera L Brazina J Svadlenka J Benesova S Soukup J. Targeting of BCL2 family proteins with ABT-199 and homoharringtonine reveals BCL2- and MCL1-dependent subgroups of diffuse large B-cell lymphoma. Clin Cancer Res. (2016) 22:1138–49. doi: 10.1158/1078-0432.CCR-15-1191 26467384

[52] Lv J Xia Q Wang J Shen Q Zhang J Zhou X. EphB4 promotes the proliferation, invasion, and angiogenesis of human colorectal cancer. Exp Mol Pathol. (2016) 100:402–8. doi: 10.1016/j.yexmp.2016.03.011 27072105

[53] Kadife E Ware T Luwor RB Chan S Nurgali K Senior PV. Effects of EphB4 receptor expression on colorectal cancer cells, tumor growth, vascularization and composition. Acta Oncol. (2018) 57:1043–56. doi: 10.1080/0284186X.2018.1429650 29368976

[54] Alzahrani AS. PI3K/Akt/mTOR inhibitors in cancer: At the bench and bedside. Semin Cancer Biol. (2019) 59:125–32. doi: 10.1016/j.semcancer.2019.07.009 31323288

[55] Ediriweera MK Tennekoon KH Samarakoon SR. Role of the PI3K/AKT/mTOR signaling pathway in ovarian cancer: Biological and therapeutic significance. Semin Cancer Biol. (2019) 59:147–60. doi: 10.1016/j.semcancer.2019.05.012 31128298

[56] Chen B Song Y Zhan Y Zhou S Ke J Ao W. Fangchinoline inhibits non-small cell lung cancer metastasis by reversing epithelial-mesenchymal transition and suppressing the cytosolic ROS-related Akt-mTOR signaling pathway. Cancer Lett. (2022) 543:215783. doi: 10.1016/j.canlet.2022.215783 35700820

[57] Shi X Yang J Liu M Zhang Y Zhou Z Luo W. Circular RNA ANAPC7 inhibits tumor growth and muscle wasting via PHLPP2-AKT-TGF-β Signaling axis in pancreatic cancer. Gastroenterology. (2022) 162:2004–17.e2. doi: 10.1053/j.gastro.2022.02.017 35176309 PMC10428768

[58] Wang D Wenjie T Xu Z Peidong Y Xin M Jia C. Blockage of mTOR signaling pathway by homoharringtonine inhibits proliferation and induces apoptosis of HT29 human colorectal tumor cells. Chin J Cell Mol Immunol. (2018) 34:346–53. 29973326

[59] Beyer K Baukloh AK Stoyanova A Kamphues C Sattler A Kotsch K. Interactions of tumor necrosis factor-related apoptosis-inducing ligand (TRAIL) with the immune system: implications for inflammation and cancer. Cancers (Basel). (2019) 11:1161. doi: 10.3390/cancers11081161 31412671 PMC6721490

[60] Suehiro Y Tsuge M Kurihara M Uchida T Fujino H Ono A. Hepatitis B virus (HBV) upregulates TRAIL-R3 expression in hepatocytes resulting in escape from both cell apoptosis and suppression of HBV replication by TRAIL. J Infect Dis. (2023) 227:686–95. doi: 10.1093/infdis/jiac044 35226068

[61] Beranova L Pombinho AR Spegarova J Koc M Klanova M Molinsky J. The plant alkaloid and anti-leukemia drug homoharringtonine sensitizes resistant human colorectal carcinoma cells to TRAIL-induced apoptosis via multiple mechanisms. Apoptosis. (2013) 18:739–50. doi: 10.1007/s10495-013-0823-9 23456623

[62] Vinarsky V Krivanek J Rankel L Nahacka Z Barta T Jaros J. Human embryonic and induced pluripotent stem cells express TRAIL receptors and can be sensitized to TRAIL-induced apoptosis. Stem Cells Dev. (2013) 22:2964–74. doi: 10.1089/scd.2013.0057 PMC3822377 23806100

[63] Philipp S Sosna J Plenge J Kalthoff H Adam D. Homoharringtonine, a clinically approved anti-leukemia drug, sensitizes tumor cells for TRAIL-induced necroptosis. Cell Commun Signal. (2015) 13:25. doi: 10.1186/s12964-015-0103-0 25925126 PMC4411737

[64] Yang C Zhang H Chen M Wang S Qian R Zhang L. A survey of optimal strategy for signature-based drug repositioning and an application to liver cancer. Elife. (2022) 11:e71880. doi: 10.7554/eLife.71880 35191375 PMC8893721

[65] Camargo FD Gokhale S Johnnidis JB Fu D Bell GW Jaenisch R. YAP1 increases organ size and expands undifferentiated progenitor cells. Curr Biol. (2007) 17:2054–60. doi: 10.1016/j.cub.2007.10.039 17980593

[66] Wang H Wang R Huang D Li S Gao B Kang Z. Homoharringtonine exerts anti-tumor effects in hepatocellular carcinoma through activation of the hippo pathway. Front Pharmacol. (2021) 12:592071. doi: 10.3389/fphar.2021.592071 33716735 PMC7943857

[67] Bure IV Nemtsova MV Zaletaev DV. Roles of E-cadherin and noncoding RNAs in the epithelial-mesenchymal transition and progression in gastric cancer. Int J Mol Sci. (2019) 20:2870. doi: 10.3390/ijms20122870 31212809 PMC6627057

[68] Zhou P Li Y Li B Zhang M Liu Y Yao Y. NMIIA promotes tumor growth and metastasis by activating the Wnt/β-catenin signaling pathway and EMT in pancreatic cancer. Oncogene. (2019) 38:5500–15. doi: 10.1038/s41388-019-0806-6 30967633

[69] Navarini NF De Araújo VC Sperandio M Napimoga MH Teixeira LN De Araújo NS. Effect of epithelial growth factor on matrix metalloproteinase-2 and E-cadherin/β-catenin expression in an in *situ* model of tumorigenesis. Oncol Lett. (2017) 14:3136–40. doi: 10.3892/ol.2017.6513 PMC5588072 28927057

[70] Zhu M Gong Z Wu Q Su Q Yang T Yu R. Homoharringtonine suppresses tumor proliferation and migration by regulating EphB4-mediated β-catenin loss in hepatocellular carcinoma. Cell Death Dis. (2020) 11:632. doi: 10.1038/s41419-020-02902-2 32801343 PMC7429962

[71] Liu HY Dong TX Li ZZ Li TT Jiang J Zhu MW. Homoharringtonine inhibits the progression of hepatocellular carcinoma by suppressing the PI3K/AKT/GSK3β/Slug signaling pathway. Neoplasma. (2021) 68:924–37. doi: 10.4149/neo_2021_210113N57 33998239

[72] Bednarski JJ Sleckman BP. Integrated signaling in developing lymphocytes: the role of DNA damage responses. Cell Cycle. (2012) 11:4129–34. doi: 10.4161/cc.22021 PMC3524208 23032308

[73] Yumei D Jiafeng T Shiying H Tao Z Ruijin L Shuang HE. Homoharringtonine activates ATM/p53 pathway to inhibit proliferation of human liver cancer cell PLC5. Chin Pharmacol Bull. (2021) 37:380–5. doi: 10.3969/j.issn.1001-1978.2021.03.015

[74] He F Ru X Wen T. NRF2, a transcription factor for stress response and beyond. Int J Mol Sci. (2020) 21:4777. doi: 10.3390/ijms21134777 32640524 PMC7369905

[75] Kang JS Lee J Nam LB Yoo OK Pham KT Duong TH. Homoharringtonine stabilizes secondary structure of guanine-rich sequence existing in the 5’-untranslated region of Nrf2. Bioorg Med Chem Lett. (2019) 29:2189–96. doi: 10.1016/j.bmcl.2019.06.049 31270017

[76] Wang H Zou L Ma K Yu J Wu H Wei M. Cell-specific mechanisms of TMEM16A Ca(2+)-activated chloride channel in cancer. Mol Cancer. (2017) 16:152. doi: 10.1186/s12943-017-0720-x 28893247 PMC5594453

[77] Duvvuri U Shiwarski DJ Xiao D Bertrand C Huang X Edinger RS. TMEM16A induces MAPK and contributes directly to tumorigenesis and cancer progression. Cancer Res. (2012) 72:3270–81. doi: 10.1158/0008-5472.CAN-12-0475-T PMC3694774 22564524

[78] Guo S Bai X Shi S Deng Y Kang X An H. TMEM16A, a homoharringtonine receptor, as a potential endogenic target for lung cancer treatment. Int J Mol Sci. (2021) 22:10930. doi: 10.3390/ijms222010930 34681590 PMC8535866

[79] Kranenburg O. The KRAS oncogene: past, present, and future. Biochim Biophys Acta. (2005) 1756:81–2. doi: 10.1016/j.bbcan.2005.10.001 16269215

[80] Roberts PJ Stinchcombe TE. KRAS mutation: should we test for it, and does it matter. J Clin Oncol. (2013) 31:1112–21. doi: 10.1200/JCO.2012.43.0454 23401440

[81] Weng TY Wu HF Li CY Hung YH Chang YW Chen YL. Homoharringtonine induced immune alteration for an Efficient Anti-tumor Response in Mouse Models of Non-small Cell Lung Adenocarcinoma Expressing Kras Mutation. Sci Rep. (2018) 8:8216. doi: 10.1038/s41598-018-26454-w 29844447 PMC5974086

[82] Yu H Lee H Herrmann A Buettner R Jove R. Revisiting STAT3 signalling in cancer: new and unexpected biological functions. Nat Rev Cancer. (2014) 14:736–46. doi: 10.1038/nrc3818 25342631

[83] Isomäki P Junttila I Vidqvist KL Korpela M Silvennoinen O. The activity of JAK-STAT pathways in rheumatoid arthritis: constitutive activation of STAT3 correlates with interleukin 6 levels. Rheumatol (Oxford). (2015) 54:1103–13. doi: 10.1093/rheumatology/keu430 25406356

[84] Cao W Liu Y Zhang R Zhang B Wang T Zhu X. Homoharringtonine induces apoptosis and inhibits STAT3 via IL-6/JAK1/STAT3 signal pathway in Gefitinib-resistant lung cancer cells. Sci Rep. (2015) 5:8477. doi: 10.1038/srep08477 26166037 PMC4499885

[85] Yakhni M Briat A El Guerrab A Furtado L Kwiatkowski F Miot-Noirault E. Homoharringtonine, an approved anti-leukemia drug, suppresses triple negative breast cancer growth through a rapid reduction of anti-apoptotic protein abundance. Am J Cancer Res. (2019) 9:1043–60. PMC6556597 31218111

[86] Shen M Dong C Ruan X Yan W Cao M Pizzo D. Chemotherapy-induced extracellular vesicle miRNAs promote breast cancer stemness by targeting ONECUT2. Cancer Res. (2019) 79:3608–21. doi: 10.1158/0008-5472.CAN-18-4055 PMC8972808 31118200

[87] Wang LB Wang DN Wu LG Cao J Tian JH Liu R. Homoharringtonine inhibited breast cancer cells growth via miR-18a-3p/AKT/mTOR signaling pathway. Int J Biol Sci. (2021) 17:995–1009. doi: 10.7150/ijbs.44907 33867824 PMC8040299

[88] Plett R Mellor P Kendall S Hammond SA Boulet A Plaza K. Homoharringtonine demonstrates a cytotoxic effect against triple-negative breast cancer cell lines and acts synergistically with paclitaxel. Sci Rep. (2022) 12:15663. doi: 10.1038/s41598-022-19621-7 36123435 PMC9485251

[89] Lei LIY ZHOU Hong L. Effects of homoharringtonine on the migration and invasion ability of human triple negative breast cancer cells. J Med Res Combat Trauma Care. (2024) 37:567–74. doi: 10.16571/j.cnki.2097-2768.2024.06.002

[90] Cooper J Giancotti FG. Integrin signaling in cancer: mechanotransduction, stemness, epithelial plasticity, and therapeutic resistance. Cancer Cell. (2019) 35:347–67. doi: 10.1016/j.ccell.2019.01.007 PMC6684107 30889378

[91] Li M Wang Y Li M Wu X Setrerrahmane S Xu H. Integrins as attractive targets for cancer therapeutics. Acta Pharm Sin B. (2021) 11:2726–37. doi: 10.1016/j.apsb.2021.01.004 PMC8463276 34589393

[92] Mitra SK Schlaepfer DD. Integrin-regulated FAK-Src signaling in normal and cancer cells. Curr Opin Cell Biol. (2006) 18:516–23. doi: 10.1016/j.ceb.2006.08.011 16919435

[93] Wu Q Chen P Li J Lin Z Zhang Q Kwok HF. Inhibition of bladder cancer growth with homoharringtonine by inactivating integrin α5/β1-FAK/Src axis: A novel strategy for drug application. Pharmacol Res. (2023) 188:106654. doi: 10.1016/j.phrs.2023.106654 36640858

[94] Jiafeng T Tao Z Shiying H Yumei D LI J Jianhua R. Effect of homoharringtonine on proliferation of human melanoma A375 cells and its mechanisms. Chin Pharmacol Bull. (2020) 36:1100–5. doi: 10.3969/j.issn.1001-1978.2020.08.013

[95] Shiying H Di Z Tao Z Jiafeng T Yumei D Rui L. Homoharringtonine inhibits the cell cycle and proliferation of vemurafenib-resistant melanoma cells by downregulating the expression of IRS4. Chin J Biochem Mol Biol. (2020) 36:970–6. doi: 10.13865/j.cnki.cjbmb.2020.05.1078

[96] Smith J Tho LM Xu N Gillespie DA. The ATM-Chk2 and ATR-Chk1 pathways in DNA damage signaling and cancer. Adv Cancer Res. (2010) 108:73–112. doi: 10.1016/B978-0-12-380888-2.00003-0 21034966

[97] Ma HT Poon R. Aurora kinases and DNA damage response. Mutat Res. (2020) 821:111716. doi: 10.1016/j.mrfmmm.2020.111716 32738522

[98] Tang JF Li GL Zhang T Du YM Huang SY Ran JH. Homoharringtonine inhibits melanoma cells proliferation *in vitro* and vivo by inducing DNA damage, apoptosis, and G2/M cell cycle arrest. Arch Biochem Biophys. (2021) 700:108774. doi: 10.1016/j.abb.2021.108774 33548212

[99] Fischer-Valuck BW Chen I Srivastava AJ Floberg JM Rao YJ King AA. Assessment of the treatment approach and survival outcomes in a modern cohort of patients with atypical teratoid rhabdoid tumors using the National Cancer Database. Cancer. (2017) 123:682–7. doi: 10.1002/cncr.30405 27861763

[100] Roberts CW Biegel JA. The role of SMARCB1/INI1 in development of rhabdoid tumor. Cancer Biol Ther. (2009) 8:412–6. doi: 10.4161/cbt.8.5.8019 PMC2709499 19305156

[101] Howard TP Oberlick EM Rees MG Arnoff TE Pham MT Brenan L. Rhabdoid tumors are sensitive to the protein-translation inhibitor homoharringtonine. Clin Cancer Res. (2020) 26:4995–5006. doi: 10.1158/1078-0432.CCR-19-2717 32631955 PMC7501142

[102] Villano JL Seery TE Bressler LR. Temozolomide in Malignant gliomas: current use and future targets. Cancer Chemother Pharmacol. (2009) 64:647–55. doi: 10.1007/s00280-009-1050-5 19543728

[103] Ball SG Shuttleworth CA Kielty CM. Platelet-derived growth factor receptor-alpha is a key determinant of smooth muscle alpha-actin filaments in bone marrow-derived mesenchymal stem cells. Int J Biochem Cell Biol. (2007) 39:379–91. doi: 10.1016/j.biocel.2006.09.005 17070723

[104] Vignais ML Sadowski HB Watling D Rogers NC Gilman M. Platelet-derived growth factor induces phosphorylation of multiple JAK family kinases and STAT proteins. Mol Cell Biol. (1996) 16:1759–69. doi: 10.1128/MCB.16.4.1759 PMC231162 8657151

[105] Bowman T Broome MA Sinibaldi D Wharton W Pledger WJ Sedivy JM. Stat3-mediated Myc expression is required for Src transformation and PDGF-induced mitogenesis. Proc Natl Acad Sci U.S.A. (2001) 98:7319–24. doi: 10.1073/pnas.131568898 PMC34666 11404481

[106] Jin Y Ding L Ding Z Fu Y Song Y Jing Y. Tensile force-induced PDGF-BB/PDGFRβ signals in periodontal ligament fibroblasts activate JAK2/STAT3 for orthodontic tooth movement. Sci Rep. (2020) 10:11269. doi: 10.1038/s41598-020-68068-1 32647179 PMC7347599

[107] Chang N Ahn SH Kong DS Lee HW Nam DH. The role of STAT3 in glioblastoma progression through dual influences on tumor cells and the immune microenvironment. Mol Cell Endocrinol. (2017) 451:53–65. doi: 10.1016/j.mce.2017.01.004 28089821

[108] Porcù E Maule F Manfreda L Mariotto E Bresolin S Cani A. Identification of Homoharringtonine as a potent inhibitor of glioblastoma cell proliferation and migration. Transl Res. (2023) 251:41–53. doi: 10.1016/j.trsl.2022.06.017 35788055

[109] Diakos CI Charles KA McMillan DC Clarke SJ. Cancer-related inflammation and treatment effectiveness. Lancet Oncol. (2014) 15:e493–503. doi: 10.1016/S1470-2045(14)70263-3 25281468

[110] Jones SA Scheller J Rose-John S. Therapeutic strategies for the clinical blockade of IL-6/gp130 signaling. J Clin Invest. (2011) 121:3375–83. doi: 10.1172/JCI57158 PMC3163962 21881215

[111] Fan Y Mao R Yang J. NF-κB and STAT3 signaling pathways collaboratively link inflammation to cancer. Protein Cell. (2013) 4:176–85. doi: 10.1007/s13238-013-2084-3 PMC4875500 23483479

[112] Revathidevi S Munirajan AK. Akt in cancer: Mediator and more. Semin Cancer Biol. (2019) 59:80–91. doi: 10.1016/j.semcancer.2019.06.002 31173856

[113] Bromberg JF Wrzeszczynska MH Devgan G Zhao Y Pestell RG Albanese C. Stat3 as an oncogene. Cell. (1999) 98:295–303. doi: 10.1016/s0092-8674(00)81959-5 10458605

[114] Wang T Fahrmann JF Lee H Li YJ Tripathi SC Yue C. JAK/STAT3-regulated fatty acid β-oxidation is critical for breast cancer stem cell self-renewal and chemoresistance. Cell Metab. (2018) 27:136–50.e5. doi: 10.1016/j.cmet.2017.11.001 29249690 PMC5777338

[115] Jiang X Wu Q Zhang C Wang M. Homoharringtonine inhibits alzheimer’s disease progression by reducing neuroinflammation via STAT3 signaling in APP/PS1 mice. Neurodegener Dis. (2021) 21:93–102. doi: 10.1159/000519974 34808617

[116] Huang Y Chen Z Wang Y Ba X Huang Y Shen P. Triptolide exerts an anti-tumor effect on non−small cell lung cancer cells by inhibiting activation of the IL−6/STAT3 axis. Int J Mol Med. (2019) 44:291–300. doi: 10.3892/ijmm.2019.4197 31115521

[117] Miagkov AV Kovalenko DV Brown CE Didsbury JR Cogswell JP Stimpson SA. NF-kappaB activation provides the potential link between inflammation and hyperplasia in the arthritic joint. Proc Natl Acad Sci U.S.A. (1998) 95:13859–64. doi: 10.1073/pnas.95.23.13859 PMC24931 9811891

[118] Yang L Cohn L Zhang DH Homer R Ray A Ray P. Essential role of nuclear factor kappaB in the induction of eosinophilia in allergic airway inflammation. J Exp Med. (1998) 188:1739–50. doi: 10.1084/jem.188.9.1739 PMC2212522 9802985

[119] Bondeson J Foxwell B Brennan F Feldmann M. Defining therapeutic targets by using adenovirus: blocking NF-kappaB inhibits both inflammatory and destructive mechanisms in rheumatoid synovium but spares anti-inflammatory mediators. Proc Natl Acad Sci U.S.A. (1999) 96:5668–73. doi: 10.1073/pnas.96.10.5668 PMC21918 10318942

[120] Donovan CE Mark DA He HZ Liou HC Kobzik L Wang Y. NF-kappa B/Rel transcription factors: c-Rel promotes airway hyperresponsiveness and allergic pulmonary inflammation. J Immunol. (1999) 163:6827–33. doi: 10.4049/jimmunol.163.12.6827 10586083

[121] Karin M Cao Y Greten FR Li ZW. NF-kappaB in cancer: from innocent bystander to major culprit. Nat Rev Cancer. (2002) 2:301–10. doi: 10.1038/nrc780 12001991

[122] Kim M Jo H Kwon Y Kim Y Jung HS Jeoung D. Homoharringtonine inhibits allergic inflammations by regulating NF-κB-miR-183-5p-BTG1 axis. Front Pharmacol. (2020) 11:1032. doi: 10.3389/fphar.2020.01032 32733254 PMC7358642

[123] Dong HJ Wang ZH Meng W Li CC Hu YX Zhou L. The natural compound homoharringtonine presents broad antiviral activity *in vitro* and *in vivo* . Viruses. (2018) 10:601. doi: 10.3390/v10110601 30388805 PMC6266276

[124] Cao J Forrest JC Zhang X. A screen of the NIH Clinical Collection small molecule library identifies potential anti-coronavirus drugs. Antiviral Res. (2015) 114:1–10. doi: 10.1016/j.antiviral.2014.11.010 25451075 PMC7113785

[125] Kim JE Song YJ. Anti-varicella-zoster virus activity of cephalotaxine esters *in vitro* . J Microbiol. (2019) 57:74–9. doi: 10.1007/s12275-019-8514-z PMC7090801 30456755

[126] Choy KT Wong AY Kaewpreedee P Sia SF Chen D Hui K. Remdesivir, lopinavir, emetine, and homoharringtonine inhibit SARS-CoV-2 replication *in vitro* . Antiviral Res. (2020) 178:104786. doi: 10.1016/j.antiviral.2020.104786 32251767 PMC7127386

[127] Ianevski A Yao R Fenstad MH Biza S Zusinaite E Reisberg T. Potential antiviral options against SARS-coV-2 infection. Viruses. (2020) 12:642. doi: 10.3390/v12060642 32545799 PMC7354438

[128] Islam MT Sarkar C El-Kersh DM Jamaddar S Uddin SJ Shilpi JA. Natural products and their derivatives against coronavirus: A review of the non-clinical and pre-clinical data. Phytother Res. (2020) 34:2471–92. doi: 10.1002/ptr.6700 32248575

[129] Ma H Wen H Qin Y Wu S Zhang G Wu CI. Homo-harringtonine, highly effective against coronaviruses, is safe in treating COVID-19 by nebulization. Sci China Life Sci. (2022) 65:1263–6. doi: 10.1007/s11427-021-2093-2 PMC8972673 35362917

[130] Shen Z Halberg A Fong JY Guo J Song G Louie B. Elucidating host cell response pathways and repurposing therapeutics for SARS-CoV-2 and other coronaviruses. Sci Rep. (2022) 12:18811. doi: 10.1038/s41598-022-21984-w 36335206 PMC9637228

[131] Su M Shi D Xing X Qi S Yang D Zhang J. Coronavirus porcine epidemic diarrhea virus nucleocapsid protein interacts with p53 to induce cell cycle arrest in S-phase and promotes viral replication. J Virol. (2021) 95:e0018721. doi: 10.1128/JVI.00187-21 34037422 PMC8373254

[132] Gray NK Wickens M. Control of translation initiation in animals. Annu Rev Cell Dev Biol. (1998) 14:399–458. doi: 10.1146/annurev.cellbio.14.1.399 9891789

[133] Panda S Vedagiri D Viveka TS Harshan KH. A unique phosphorylation-dependent eIF4E assembly on 40S ribosomes co-ordinated by hepatitis C virus protein NS5A that activates internal ribosome entry site translation. Biochem J. (2014) 462:291–302. doi: 10.1042/BJ20131530 24894874

[134] Montero H García-Román R Mora SI. eIF4E as a control target for viruses. Viruses. (2015) 7:739–50. doi: 10.3390/v7020739 PMC4353914 25690796

[135] Royall E Doyle N Abdul-Wahab A Emmott E Morley SJ Goodfellow I. Murine norovirus 1 (MNV1) replication induces translational control of the host by regulating eIF4E activity during infection. J Biol Chem. (2015) 290:4748–58. doi: 10.1074/jbc.M114.602649 PMC4335213 25561727

[136] Romero MR Serrano MA Efferth T Alvarez M Marin JJ. Effect of cantharidin, cephalotaxine and homoharringtonine on “*in vitro*” models of hepatitis B virus (HBV) and bovine viral diarrhoea virus (BVDV) replication. Planta Med. (2007) 73:552–8. doi: 10.1055/s-2007-967184 17458779

[137] Andersen PI Krpina K Ianevski A Shtaida N Jo E Yang J. Novel antiviral activities of obatoclax, emetine, niclosamide, brequinar, and homoharringtonine. Viruses. (2019) 11:964. doi: 10.3390/v11100964 31635418 PMC6832696

[138] Kaur P Thiruchelvan M Lee RC Chen H Chen KC Ng ML. Inhibition of chikungunya virus replication by harringtonine, a novel antiviral that suppresses viral protein expression. Antimicrob Agents Chemother. (2013) 57:155–67. doi: 10.1128/AAC.01467-12 PMC3535938 23275491

[139] Sun Y Ge Y Fu Y Yan L Cai J Shi K. Mitomycin C induces fibroblasts apoptosis and reduces epidural fibrosis by regulating miR-200b and its targeting of RhoE. Eur J Pharmacol. (2015) 765:198–208. doi: 10.1016/j.ejphar.2015.08.002 26254780

[140] Hu X Xu Q Wan H Hu Y Xing S Yang H. PI3K-Akt-mTOR/PFKFB3 pathway mediated lung fibroblast aerobic glycolysis and collagen synthesis in lipopolysaccharide-induced pulmonary fibrosis. Lab Invest. (2020) 100:801–11. doi: 10.1038/s41374-020-0404-9 32051533

[141] Sun Y Dai J Jiao R Jiang Q Wang J. Homoharringtonine inhibits fibroblasts proliferation, extracellular matrix production and reduces surgery-induced knee arthrofibrosis via PI3K/AKT/mTOR pathway-mediated apoptosis. J Orthop Surg Res. (2021) 16:9. doi: 10.1186/s13018-020-02150-2 33407698 PMC7789651

[142] Chao P Yiqiao X Changtai YU Anhuai Y,E. An experimental research on inhibitory effect of homoharringtonine on fibrin proliferation. China J Chin Ophthalmol. (2003) 03):10–2. doi: 10.3969/j.issn.1002-4379.2003.03.003

[143] Peng Chao WL Zhang Dingyu HY Min M. The clinical study of homenarringnine on inhibited traumatic proliferative vitreoretinopathy. China J Chin Ophthalmol. (2005) 03):135–7. doi: 10.3969/j.issn.1002-4379.2005.03.004

[144] Li JP Hu CZ Zeng SQ Ren JM Wei HR. Inhibition of intraocular proliferation by homoharringtonine. Exp study. Graefes Arch Clin Exp Ophthalmol. (1988) 226:367–70. doi: 10.1007/BF02172969 3169589

[145] Zhenmei A Songquan W Shuhui X Guizhi Z. Effects of homoharringtonine on proliferation of human retro-ocular fibroblasts. J Of West China Univ Of Med Sci. (2000) 01):95–7. 12501627

[146] Zhang M Wang L Wang Y Ding Z Mai C Nie S. Effect of homoharringtonine on the corneal haze after excimer laser photorefractive keratectomy in rabbits. J Huazhong Univ Sci Technolog Med Sci. (2005) 25:289–92. doi: 10.1007/BF02896186 16696341

[147] Baowen G Jianrong HU Zonghui Y Mian S. Hemoharringtonine in preventing Haze after PRK. Int Eye Sci. (2005) 04):683–4. doi: 10.3969/j.issn.1672-5123.2005.04.022

[148] Keming Y Dawei P Xing L Xiang T. A clinial trial of homoharringtonine as an adjunctive agent of filtering surgery in patients with refractory glaucoma. China J Chin Ophthalmol. (2001) 04):19–22. doi: 10.3969/j.issn.1002-4379.2001.04.008

[149] Leng F Edison P. Neuroinflammation and microglial activation in Alzheimer disease: where do we go from here. Nat Rev Neurol. (2021) 17:157–72. doi: 10.1038/s41582-020-00435-y 33318676

[150] Noh K Kim M Kim Y Kim H Kim H Byun J. miR-122-SOCS1-JAK2 axis regulates allergic inflammation and allergic inflammation-promoted cellular interactions. Oncotarget. (2017) 8:63155–76. doi: 10.18632/oncotarget.19149 PMC5609911 28968979

[151] Yu W Mao L Qian J Qian W Meng H Mai W. Homoharringtonine in combination with cytarabine and aclarubicin in the treatment of refractory/relapsed acute myeloid leukemia: a single-center experience. Ann Hematol. (2013) 92:1091–100. doi: 10.1007/s00277-013-1758-5 23595277

[152] Lam SS Ho ES He BL Wong WW Cher CY Ng NK. Homoharringtonine (omacetaxine mepesuccinate) as an adjunct for FLT3-ITD acute myeloid leukemia. Sci Transl Med. (2016) 8:359ra129. doi: 10.1126/scitranslmed.aaf3735 27708062

[153] Jin H Zhang Y Yu S Du X Xu N Shao R. Venetoclax combined with azacitidine and homoharringtonine in relapsed/refractory AML: A multicenter, phase 2 trial. J Hematol Oncol. (2023) 16:42. doi: 10.1186/s13045-023-01437-1 37120593 PMC10149010

[154] Li Y Deng Z Zho J Ding B Shi Y Li Y. Homoharringtonine combined with cytarabine to treat chronic myelogenous leukemia in myeloid blast crisis and its impact on bone marrow CD34+CD7+ cells. Acta Haematol. (2014) 132:172–6. doi: 10.1159/000356742 24603361

[155] Kantarjian HM Talpaz M Smith TL Cortes J Giles FJ Rios MB. Homoharringtonine and low-dose cytarabine in the management of late chronic-phase chronic myelogenous leukemia. J Clin Oncol. (2000) 18:3513–21. doi: 10.1200/JCO.2000.18.20.3513 11032593

[156] O’Brien S Giles F Talpaz M Cortes J Rios MB Shan J. Results of triple therapy with interferon-alpha, cytarabine, and homoharringtonine, and the impact of adding imatinib to the treatment sequence in patients with Philadelphia chromosome-positive chronic myelogenous leukemia in early chronic phase. Cancer. (2003) 98:888–93. doi: 10.1002/cncr.11620 12942553

[157] Quintás-Cardama A Kantarjian H Garcia-Manero G O’Brien S Faderl S Estrov Z. Phase I/II study of subcutaneous homoharringtonine in patients with chronic myeloid leukemia who have failed prior therapy. Cancer. (2007) 109:248–55. doi: 10.1002/cncr.22398 17154172

[158] Stone RM Donohue KA Stock W Hars V Linker CA Shea T. A phase II study of continuous infusion homoharringtonine and cytarabine in newly diagnosed patients with chronic myeloid leukemia: CALGB study 19804. Cancer Chemother Pharmacol. (2009) 63:859–64. doi: 10.1007/s00280-008-0805-8 18670778

[159] Huang BT Zeng QC Yu J Liu XL Xiao Z Zhu HQ. High-dose homoharringtonine versus standard-dose daunorubicin is effective and safe as induction and post-induction chemotherapy for elderly patients with acute myeloid leukemia: a multicenter experience from China. Med Oncol. (2012) 29:251–9. doi: 10.1007/s12032-011-9820-4 21258877

[160] Feldman E Arlin Z Ahmed T Mittelman A Puccio C Chun H. Homoharringtonine in combination with cytarabine for patients with acute myelogenous leukemia. Leukemia. (1992) 6:1189–91. 1434803

[161] Feldman E Arlin Z Ahmed T Mittelman A Puccio C Chun H. Homoharringtonine is safe and effective for patients with acute myelogenous leukemia. Leukemia. (1992) 6:1185–8. 1434802

[162] Bell BA Chang MN Weinstein HJ. A phase II study of Homoharringtonine for the treatment of children with refractory or recurrent acute myelogenous leukemia: a pediatric oncology group study. Med Pediatr Oncol. (2001) 37:103–7. doi: 10.1002/mpo.1177 11496347

[163] Zhang JY Yu K Li LJ. Comparison of efficacy of HCAG and FLAG re-induction chemotherapy in acute myeloid leukemia patients of low- and intermediate-risk groups. Clin Transl Oncol. (2019) 21:1543–50. doi: 10.1007/s12094-019-02085-z 30915633

[164] Zhang C Lam S Leung G Tsui SP Yang N Ng N. Sorafenib and omacetaxine mepesuccinate as a safe and effective treatment for acute myeloid leukemia carrying internal tandem duplication of Fms-like tyrosine kinase 3. Cancer. (2020) 126:344–53. doi: 10.1002/cncr.32534 31580501

[165] Zhang JY Li L Liu W Jin Y Zhao M Zhou Y. Comparison of efficacy of HCAG and CAG re-induction chemotherapy in elderly low- and intermediate-risk group patients diagnosed with acute myeloid leukemia. Clin Transl Oncol. (2021) 23:48–57. doi: 10.1007/s12094-020-02383-x 32458310

[166] Zhang Y Li X Weng X Shen Y Chen Y Zheng Y. Optimization of idarubicin and cytarabine induction regimen with homoharringtonine for newly diagnosed acute myeloid leukemia patients based on the peripheral blast clearance rate: A single-arm, phase 2 trial (RJ-AML 2014). Am J Hematol. (2022) 97:43–51. doi: 10.1002/ajh.26386 34687467

[167] Yu S Zhang Y Yu G Wang Y Shao R Du X. Sorafenib plus triplet therapy with venetoclax, azacitidine and homoharringtonine for refractory/relapsed acute myeloid leukemia with FLT3-ITD: A multicenter phase 2 study. J Intern Med. (2024) 295:216–28. doi: 10.1111/joim.13738 37899297

[168] Jin J Wang JX Chen FF Wu DP Hu J Zhou JF. Homoharringtonine-based induction regimens for patients with de-novo acute myeloid leukaemia: a multicentre, open-label, randomised, controlled phase 3 trial. Lancet Oncol. (2013) 14:599–608. doi: 10.1016/S1470-2045(13)70152-9 23664707

[169] Marin D Kaeda JS Andreasson C Saunders SM Bua M Olavarria E. Phase I/II trial of adding semisynthetic homoharringtonine in chronic myeloid leukemia patients who have achieved partial or complete cytogenetic response on imatinib. Cancer. (2005) 103:1850–5. doi: 10.1002/cncr.20975 15786422

[170] Li YF Deng ZK Xuan HB Zhu JB Ding BH Liu XN. Prolonged chronic phase in chronic myelogenous leukemia after homoharringtonine therapy. Chin Med J (Engl). (2009) 122:1413–7. 19567163

[171] Li YF Deng Z Din BH Zhu JB. Effect of homoharringtonine on bone marrow CD34 + CD117 + cells in patients with chronic myelogenous leukemia. Leuk Lymphoma. (2012) 53:934–9. doi: 10.3109/10428194.2011.635859 22054289

[172] Cortes JE Nicolini FE Wetzler M Lipton JH Akard L Craig A. Subcutaneous omacetaxine mepesuccinate in patients with chronic-phase chronic myeloid leukemia previously treated with 2 or more tyrosine kinase inhibitors including imatinib. Clin Lymphoma Myeloma Leuk. (2013) 13:584–91. doi: 10.1016/j.clml.2013.03.020 PMC3775895 23787123

[173] Cortes J Digumarti R Parikh PM Wetzler M Lipton JH Hochhaus A. Phase 2 study of subcutaneous omacetaxine mepesuccinate for chronic-phase chronic myeloid leukemia patients resistant to or intolerant of tyrosine kinase inhibitors. Am J Hematol. (2013) 88:350–4. doi: 10.1002/ajh.23408 PMC5558840 23468307

[174] Cortes J Lipton JH Rea D Digumarti R Chuah C Nanda N. Phase 2 study of subcutaneous omacetaxine mepesuccinate after TKI failure in patients with chronic-phase CML with T315I mutation. Blood. (2012) 120:2573–80. doi: 10.1182/blood-2012-03-415307 PMC4916583 22896000

[175] Khoury HJ Cortes J Baccarani M Wetzler M Masszi T Digumarti R. Omacetaxine mepesuccinate in patients with advanced chronic myeloid leukemia with resistance or intolerance to tyrosine kinase inhibitors. Leuk Lymphoma. (2015) 56:120–7. doi: 10.3109/10428194.2014.889826 24650054

[176] Short NJ Jabbour E Naqvi K Patel A Ning J Sasaki K. A phase II study of omacetaxine mepesuccinate for patients with higher-risk myelodysplastic syndrome and chronic myelomonocytic leukemia after failure of hypomethylating agents. Am J Hematol. (2019) 94:74–9. doi: 10.1002/ajh.25318 PMC6570401 30328139

[177] Feldman EJ Seiter KP Ahmed T Baskind P Arlin ZA. Homoharringtonine in patients with myelodysplastic syndrome (MDS) and MDS evolving to acute myeloid leukemia. Leukemia. (1996) 10:40–2. 8558935

[178] Daver N Vega-Ruiz A Kantarjian HM Estrov Z Ferrajoli A Kornblau S. A phase II open-label study of the intravenous administration of homoharringtonine in the treatment of myelodysplastic syndrome. Eur J Cancer Care (Engl). (2013) 22:605–11. doi: 10.1111/ecc.12065 PMC3745781 23701251

[179] Wu L Li X Su J Chang C He Q Zhang X. Effect of low-dose cytarabine, homoharringtonine and granulocyte colony-stimulating factor priming regimen on patients with advanced myelodysplastic syndrome or acute myeloid leukemia transformed from myelodysplastic syndrome. Leuk Lymphoma. (2009) 50:1461–7. doi: 10.1080/10428190903096719 19672772

[180] Kaiqi L Hui W Huijun W Chunlin Z Bingcheng L Dong L. Comparison of homoharringtonine and daunorubicin cardiotoxicity in adults with acute promyelocytic leukemia. J Clin Med Pract. (2012) 16:10–2 + 17. doi: 10.3969/j.issn.1672-2353.2012.03.003

[181] Watari A Hashegawa M Yagi K Kondoh M. Homoharringtonine increases intestinal epithelial permeability by modulating specific claudin isoforms in Caco-2 cell monolayers. Eur J Pharm Biopharm. (2015) 89:232–8. doi: 10.1016/j.ejpb.2014.12.012 25513955

[182] Watari A Fujiwara K Yagi K Tachibana K Katsurada T Myoui A. Homoharringtonine is a transdermal granular permeation enhancer. Biochem Biophys Res Commun. (2022) 616:140–4. doi: 10.1016/j.bbrc.2022.04.067 35679696

[183] Yan D Wei H Lai X Ge Y Xu S Meng J. Co-delivery of homoharringtonine and doxorubicin boosts therapeutic efficacy of refractory acute myeloid leukemia. J Control Release. (2020) 327:766–78. doi: 10.1016/j.jconrel.2020.09.031 32949646

[184] Li M Fei X Shi F Dou J Wu S Wu D. Homoharringtonine delivered by high proportion PEG of long- circulating liposomes inhibits RPMI8226 multiple myeloma cells *in vitro* and *in vivo* . Am J Transl Res. (2016) 8:1355–68. PMC4859624 27186264

[185] Chen M Xiong F Ma L Yao H Wang Q Wen L. Inhibitory effect of magnetic Fe(3)O(4) nanoparticles coloaded with homoharringtonine on human leukemia cells *in vivo* and *in vitro* . Int J Nanomedicine. (2016) 11:4413–22. doi: 10.2147/IJN.S105543 PMC5019325 27660436

[186] Liu D Xing J Xiong F Yang F Gu N. Preparation and *in vivo* safety evaluations of antileukemic homoharringtonine-loaded PEGylated liposomes. Drug Dev Ind Pharm. (2017) 43:652–60. doi: 10.1080/03639045.2016.1275670 28005445

[187] Liu Q Luo L Gao X Zhang D Feng X Yang P. Co-delivery of daunorubicin and homoharringtonine in folic acid modified-liposomes for enhancing therapeutic effect on acute myeloid leukemia. J Pharm Sci. (2023) 112:123–31. doi: 10.1016/j.xphs.2022.04.014 35469834

[188] Wu J Wei B Shi Y Lu X Ding Y Wang C. Homoharringtonine enhances the effect of imatinib on chronic myelogenous leukemia cells by downregulating ZFX. Mol Med Rep. (2019) 20:3233–9. doi: 10.3892/mmr.2019.10539 PMC6755169 31432109

[189] Wang Q Ding W Wu JJ Ding YH Li YF. Effects of homoharringtonine combined with imatinib on K562 cell apoptosis and BCL6 expression. Zhongguo Shi Yan Xue Ye Xue Za Zhi. (2016) 24:1716–20. doi: 10.7534/j.issn.1009-2137.2016.06.018 28024482

[190] Wu JJ Ding YH Deng ZK Shi YY Lu XY Li YF. Effect of homoharringtonine combined with imatinib on the K562/G01 cells and its mechanism. Zhongguo Shi Yan Xue Ye Xue Za Zhi. (2017) 25:80–4. doi: 10.7534/j.issn.1009-2137.2017.01.013 28245379

[191] Huang BT Zeng QC Zhao WH Tan Y. Homoharringtonine contributes to imatinib sensitivity by blocking the EphB4/RhoA pathway in chronic myeloid leukemia cell lines. Med Oncol. (2014) 31:836. doi: 10.1007/s12032-013-0836-9 24415355

[192] Rossari F Minutolo F Orciuolo E. Past, present, and future of Bcr-Abl inhibitors: from chemical development to clinical efficacy. J Hematol Oncol. (2018) 11:84. doi: 10.1186/s13045-018-0624-2 29925402 PMC6011351

[193] Li S Bo Z Jiang Y Song X Wang C Tong Y. Homoharringtonine promotes BCR−ABL degradation through the p62−mediated autophagy pathway. Oncol Rep. (2020) 43:113–20. doi: 10.3892/or.2019.7412 PMC6908937 31789418

[194] Hayakawa F Towatari M Kiyoi H Tanimoto M Kitamura T Saito H. Tandem-duplicated Flt3 constitutively activates STAT5 and MAP kinase and introduces autonomous cell growth in IL-3-dependent cell lines. Oncogene. (2000) 19:624–31. doi: 10.1038/sj.onc.1203354 10698507

[195] Choudhary C Brandts C Schwable J Tickenbrock L Sargin B Ueker A. Activation mechanisms of STAT5 by oncogenic Flt3-ITD. Blood. (2007) 110:370–4. doi: 10.1182/blood-2006-05-024018 17356133

[196] Wang F Huang J Guo T Zheng Y Zhang L Zhang D. Homoharringtonine synergizes with quizartinib in FLT3-ITD acute myeloid leukemia by targeting FLT3-AKT-c-Myc pathway. Biochem Pharmacol. (2021) 188:114538. doi: 10.1016/j.bcp.2021.114538 33831397

[197] Huang S Pan J Jin J Li C Li X Huang J. Abivertinib, a novel BTK inhibitor: Anti-Leukemia effects and synergistic efficacy with homoharringtonine in acute myeloid leukemia. Cancer Lett. (2019) 461:132–43. doi: 10.1016/j.canlet.2019.07.008 31310800

[198] Wu Z Zhuang H Yu Q Zhang X Jiang X Lu X. Homoharringtonine combined with the heat shock protein 90 inhibitor IPI504 in the treatment of FLT3-ITD acute myeloid leukemia. Transl Oncol. (2019) 12:801–9. doi: 10.1016/j.tranon.2019.02.016 PMC6449739 30953928

[199] Shi Y Xu D Xu Y Shen H Zhang Y Ye X. Synergistic lethality effects of apatinib and homoharringtonine in acute myeloid leukemia. J Oncol. (2022) 2022:9005804. doi: 10.1155/2022/9005804 36081666 PMC9448536

[200] Cai J Huang H Hu X Lang W Fu W Xu L. Homoharringtonine synergized with gilteritinib results in the downregulation of myeloid cell leukemia-1 by upregulating UBE2L6 in FLT3-ITD-mutant acute myeloid (Leukemia) cell lines. J Oncol. (2021) 2021:3766428. doi: 10.1155/2021/3766428 34594375 PMC8478557

[201] Czabotar PE Lessene G Strasser A Adams JM. Control of apoptosis by the BCL-2 protein family: implications for physiology and therapy. Nat Rev Mol Cell Biol. (2014) 15:49–63. doi: 10.1038/nrm3722 24355989

[202] Delbridge AR Grabow S Strasser A Vaux DL. Thirty years of BCL-2: translating cell death discoveries into novel cancer therapies. Nat Rev Cancer. (2016) 16:99–109. doi: 10.1038/nrc.2015.17 26822577

[203] Shi Y Ye J Yang Y Zhao Y Shen H Ye X. The basic research of the combinatorial therapy of ABT-199 and homoharringtonine on acute myeloid leukemia. Front Oncol. (2021) 11:692497. doi: 10.3389/fonc.2021.692497 34336680 PMC8317985

[204] Wei W Huang S Ling Q Mao S Qian Y Ye W. Homoharringtonine is synergistically lethal with BCL-2 inhibitor APG-2575 in acute myeloid leukemia. J Transl Med. (2022) 20:299. doi: 10.1186/s12967-022-03497-2 35794605 PMC9258085

[205] Rejeski K Duque-Afonso J Lübbert M. AML1/ETO and its function as a regulator of gene transcription via epigenetic mechanisms. Oncogene. (2021) 40:5665–76. doi: 10.1038/s41388-021-01952-w PMC8460439 34331016

[206] Cao J Feng H Ding NN Wu QY Chen C Niu MS. Homoharringtonine combined with aclarubicin and cytarabine synergistically induces apoptosis cells and triggers caspase-3-mediated cleavage of the AML1-ETO oncoprotein. Cancer Med. (2016) 5:3205–13. doi: 10.1002/cam4.913 PMC5119976 27709797

[207] Zhang W Lu Y Zhen T Chen X Zhang M Liu P. Homoharringtonine synergy with oridonin in treatment of t (8, 21) acute myeloid leukemia. Front Med. (2019) 13:388–97. doi: 10.1007/s11684-018-0624-1 30206768

[208] Xin LI Jiayan L Xiaohong Y Zhenxing L Yong WU. Effects of triptolide combined with homoharringtonine on proliferation and apoptosis of KG-1α Cells. J Exp Hematol. (2018) 26:347–53. doi: 10.7534/j.issn.1009-2137.2018.02.007 29665897

[209] Ping JX CHEN Pei-Dong JQ YOU YUAN Qin HH. Combination of homoharringtonine with arsenic trioxide induces apoptosis of human acute myeloid leukemia cell line U937. J Exp Hematol. (2016) 24:1649–53. doi: 10.7534/j.issn.1009-2137.2016.06.007 28024471

[210] Chen P Zhan W Wang B You P Jin Q Hou D. Homoharringtonine potentiates the antileukemic activity of arsenic trioxide against acute myeloid leukemia cells. Exp Cell Res. (2019) 376:114–23. doi: 10.1016/j.yexcr.2019.02.008 30763586

[211] Tan M Zhang Q Yuan X Chen Y Wu Y. Synergistic killing effects of homoharringtonine and arsenic trioxide on acute myeloid leukemia stem cells and the underlying mechanisms. J Exp Clin Cancer Res. (2019) 38:308. doi: 10.1186/s13046-019-1295-8 31307525 PMC6631946

[212] Qiaoyan H Yi F Lan T Jinsong Y Lingyu W Haoyue C. Study on the effect and mechanism of HL-60 cell apoptosis induced by matrine combined with homoharringtonine. Chin J Hematol. (2015) 36:433–5. doi: 10.3760/cma.j.issn.0253-2727.2015.05.018 PMC7342580 26031535

[213] Yanling R Hongyan T. Effect of homoharringtonine combined with AG490 on JAK2-STAT5 associated signal pathway in HEL cells. J Exp Hematol. (2011) 19:1117–20. 22040954

[214] Quintás-Cardama A Cortes J. Omacetaxine mepesuccinate–a semisynthetic formulation of the natural antitumoral alkaloid homoharringtonine, for chronic myelocytic leukemia and other myeloid Malignancies. IDrugs. (2008) 11:356–72. 18465678

[215] Lévy V Zohar S Bardin C Vekhoff A Chaoui D Rio B. A phase I dose-finding and pharmacokinetic study of subcutaneous semisynthetic homoharringtonine (ssHHT) in patients with advanced acute myeloid leukaemia. Br J Cancer. (2006) 95(3):253–9. doi: 10.1038/sj.bjc.6603265 PMC2360653 16847470

[216] Wang AM Qiu R Zhang D Zhao XY. Therapeutic effects of an innovative BS-HH-002 drug on pancreatic cancer cells via induction of complete MCL-1 degradation. Transl Oncol. (2022) 15:101288. doi: 10.1016/j.tranon.2021.101288 34847421 PMC8633684

